# COVID-19 in Memes: The Adaptive Response of Societies to the Pandemic?

**DOI:** 10.3390/ijerph191912969

**Published:** 2022-10-10

**Authors:** Piotr Skórka, Beata Grzywacz, Dawid Moroń, Magdalena Lenda

**Affiliations:** 1 Institute of Nature Conservation, Polish Academy of Sciences, 31-120 Kraków, Poland; 2 Institute of Systematics and Evolution of Animals, Polish Academy of Sciences, 31-016 Kraków, Poland

**Keywords:** COVID-19, entertainment, extreme negative emotions, the internet, healing, health, humor, mental health, recovery, stress

## Abstract

COVID-19 expanded rapidly throughout the world, with enormous health, social, and economic consequences. Mental health is the most affected by extreme negative emotions and stress, but it has been an underestimated part of human life during the pandemic. We hypothesized that people may have responded to the pandemic spontaneously with increased interest in and creation of funny internet memes. Using Google and Google Trends, we revealed that the number of and interest in funny internet memes related to COVID-19 exploded during the spring 2020 lockdown. The interest in coronavirus memes was positively correlated with interest in mortality due to COVID-19 on a global scale, and positively associated with the real number of deaths and cases reported in different countries. We compared content of a random sample of 200 coronavirus memes with a random sample of 200 non-coronavirus memes found on the Internet. The sentiment analysis showed that coronavirus memes had a similar proportion of positive and negative words compared to non-coronavirus memes. However, an internet questionnaire revealed that coronavirus memes gained higher funniness scores than a random sample of non-coronavirus memes. Our results confirm that societies may have turned to humor to cope with the threat of SARS-CoV-2.

## 1. Introduction

The COVID-19 pandemic has had a lasting impact worldwide [1,2,3]. Emotional and behavioral responses to this ongoing crisis are multifaceted [4]. Humans experienced elevated fear and stress about SARS-CoV-2 [5,6]. The economic consequences of the pandemic have further deteriorated psychiatric states [7,8]. The experience of life-threatening and stressful events during a pandemic can modify individual behavior [6,9]. Moreover, the need to stay at home due to the perceived risk of COVID-19 limited not only the possibility of maintaining interpersonal contacts, but also reduced the possibility of engaging in entertainment, physical activity, and contact with nature, which negatively affects mental well-being [10].

The most widely known consequence of exposure to prolonged traumatic and stressful events is post-traumatic stress disorder [11,12]. A study in Germany showed that the prevalence of mental disorders was much higher than usual, with 50.6% of investigated people expressing at least one mental disorder during the COVID-19 lockdown [13]. The stress associated with the pandemic increased the level of cortisol, which has a significant negative effect on the survival of patients with COVID-19 [14,15]. Infection with SARS-CoV-2 also has acute and chronic neurological consequences [16], which may deepen the trauma and stress associated with the infection. Thus, to successfully manage COVID-19 and its aftermath, it is necessary to provide a roadmap of health-protective behaviors that can ensure the mental health of individuals and communities [4].

Clinical research indicates that the effects of traumatic events and chronic stress can be significantly buffered by humor [17,18]. Humor has been found to decrease negative emotions, increase positive feelings, and enhance distance from adversity [19]. A review of the literature on the nature of humor revealed its ability to diffuse stressful situations and reactions [20]. It is suggested that despite the fact that humor is often underappreciated and ignored in the therapeutic process, it can be a powerful healing tool [20,21]. People with a greater propensity for coping using humor in stressful situations show increased levels of salivary immunoglobulin A (S-IgA), a vital immune system protein, which is the body’s first line of defense against respiratory illnesses [22]. In addition, trauma survivors’ humor can be used to assist them in mitigating the intensity of their traumatic stress reactions, which may be especially valuable during the pandemic and while coping with infection [20,23,24,25]. Indeed, several studies demonstrated that humor may be a method of coping with traumatic events and anxiety associated with the COVID-19 pandemic. A study in Turkey showed that COVID-19-related fear is a powerful predictor of increased hopelessness [24]. However, this predictive relationship was partially buffered by humor, which may be a coping strategy [24]. In addition, during the COVID-19 outbreak in Italy, healthcare workers who reported higher use of humor-based coping strategies perceived the situation as less stressful than those who reported less use of coping humor [26]. Humor was associated with significantly lower anxiety levels in nursing students during the national lockdown in Israel [27]. However, applying humor as a coping strategy has many caveats and requires good recognition of the characteristics (e.g., age and gender) of the target group because certain forms of humor, also related to COVID-19, may be more aversive than funny [28,29].

Humor may be shared and provided in various ways [29,30]. However, the Internet may play a key role in providing and spreading humorous content while in isolation and with reduced sociality, as in the lockdown [31]. It has already been proven that users of social media (e.g., Facebook, Instagram, Snapchat, Twitter, or TikTok) have spread and commented on different information about the pandemic [32,33], thus creating a social network for the topic [34]. Moreover, social media on the Internet provides people with opportunities, as well as challenges, to co-construct entertainment and social environments tailored to their own needs, beliefs, and interests [35,36,37]. One of the most interesting ways of commenting on reality is to depict it in the form of a meme.

The term *meme* was coined by Dawkins [38], who proposed an evolutionary model of cultural change and development grounded in the replication of ideas and knowledge through imitation and cultural transfer. The concept was later adopted by internet users, and it generally describes the rapid uptake and spread of particular ideas presented as images, written text, movies, or other media on the Internet that go viral [39,40,41]. The creation of memes involves multimodal interactions between textual and visual elements [40,42]. Specifically, combinations of unexpected visual elements and semiotic extensions of a non-standard catchphrase, wordplay (e.g., paronymy), and establishing incongruity generate humorous meanings [37,42,43,44]. Relevance theory [45] stresses that there is a gap between what is literally said (or written), i.e., what is coded, and what is intended and eventually interpreted [44]. Thus, when faced with a meme, the user also has to make inferential hypotheses concerning the role of both the image and the text in the overall comprehension of the meme [43,46]. Memes are oriented to humor, but nonhumorous memes can also be found [39,40,47]. Memes often burst onto the Internet as a response to current events that are important to society [36,48,49]. Memes may diminish or augment the value of the commented phenomenon or authority [49,50]. The essential feature of memes is their ability to be replicated by user-induced mutations [39,49]. Some memes become viral and gain enormous popularity compared to other memes [43,51]. Viral memes usually differ by two or more orders of magnitude in popularity from non-viral memes [51,52]. Memes are, thus, similar to the spread of viral diseases [53]. Therefore, the inflow of new memes and meme propagation can mirror the spread of real coronavirus data.

As COVID-19 erupted, memes related to the pandemic also became widespread on social media [46,52]. COVID-19 memes are often regarded as a kind of dark humor [29] inspired by and produced in the context of grave events and topics, notably, death and illnesses [54]. As memes may be a vehicle for social bonding [36], they may also act as a collective coping strategy for alleviating the negative effects of the pandemic [55,56].

In this study, we tested the hypothesis that the COVID-19 pandemic, especially during the spring 2020 lockdown when the first worldwide measures for stopping the virus occurred, is associated with the rise in the number and interest in funny internet memes related to COVID-19. We used a definition of *coronavirus memes*: Digital images (often containing text) created for the purpose of communication about COVID-19. Furthermore, we tested the hypothesis that interest in coronavirus memes is a response to perceived risk of death from COVID-19. Thus, we expected interest in the coronavirus memes in different countries, measured using Google Trends, to be positively correlated with (1) interest in deaths due to COVID-19 and (2) pandemic statistics, such as the number of cases and deaths reported in a given country. Finally, we compared the image and text contents and valued the funniness of a random sample of coronavirus memes with a sample of internet memes unrelated to the pandemic. We expected that (3) coronavirus memes would be rated as being more humorous than random non-coronavirus memes, to be explained by differences in the image and text content.

## 2. Materials and Methods

### 2.1. Study Organization and Eligibility Criteria

**Figure 1:**
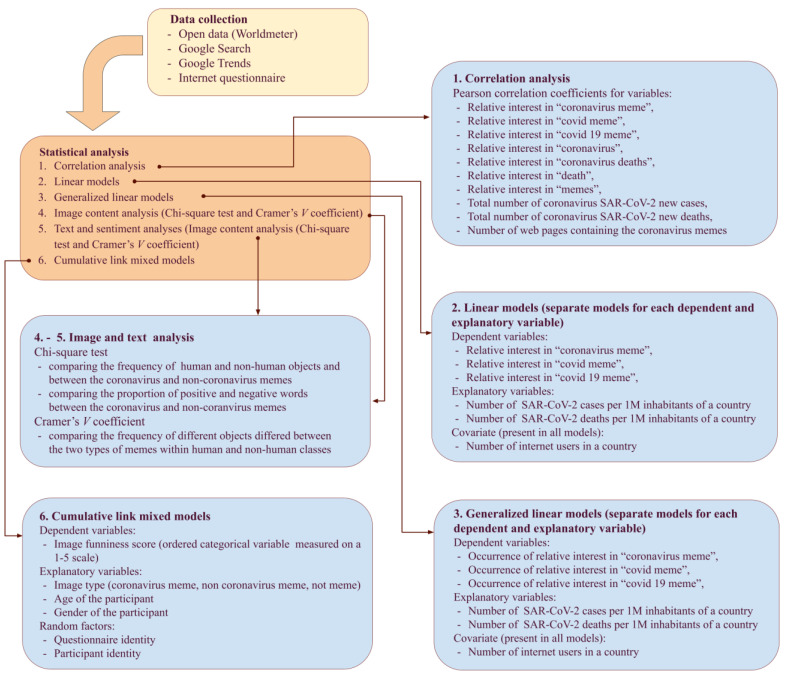
A scheme of data collection and statistical analyses used in this study.

The collected data were obtained from Google searches, Google Trends, and an internet questionnaire (Figure 1). Search in Google browser gives the estimation of the number of webpages and Google Trends gives the index on relative interest in webpages containing specified phrases. Global and country-level COVID-19 data were downloaded from Worldometer “https://www.worldometers.info/coronavirus/ (accessed on 13 October 2020)” (Figure 1). In our paper, we refer to the spring 2020 lockdown and set its boundaries between 01 March and 15 June 2020, based on data from Wehenkel [57].

First, we counted the number of webpages containing coronavirus memes from a Google search (in Google internet browser) using the search string “(coronavirus OR covid 19 OR covid) meme” (search performed on 13 October 2020 and repeated for revision purposes on 10 July 2021, with search time restricted to 13 October 2020 and before). We also constructed a time-series (at a weekly frequency from October 2019 to October 2020) representing the intensity of the meme-generating process. We performed an analogous search for non-coronavirus memes (memes not related to the COVID-19 pandemic). We did this using the search string “meme -coronavirus – covid -‘covid 19′”. We used two filters officially advised by Google Help “https://www.google.com/support/enterprise/static/gsa/docs/admin/current/gsa_doc_set/xml_reference/index.html (accessed on 10 May 2020)”. The first was the rc = 1 request parameter to request an accurate result count for up to one million documents. The second filter was automatic filtering set to 1, allowing directory filtering (filtering documents coming from the same source folder) and duplicate snippet filtering (if two documents have the same snippet generated, they will be filtered). Both filters were officially advised by Google (see [41] for details).

We used Google Trends to measure how often a particular search item was searched using Google browser relative to the total search volume. We entered the following phrases: “coronavirus meme”, “covid meme”, “covid 19 meme”, “meme”, “coronavirus”, “coronavirus death”, “death”. These strings were selected to associate the interest in coronavirus memes with interest in the coronavirus (“coronavirus”) and risk of death due to COVID-19 (“coronavirus death”) and the reference words (“death”, “meme”). The time-series obtained from this tool are informative for determining the dynamics of society’s demand for information [58] and can be a measurement of interest in a topic (e.g., [41,59]). The interest of society measured by Google Trends allows the avoidance of typical biases from questionnaires, such as dishonesty or the effect of the experimenter [41]. We analyzed the variance among different countries in the interest in coronavirus memes. The Google Trends index can be split by country-level data. In particular, we compared the relative interest in coronavirus memes with the total number of deaths due to COVID-19 and cases per one million inhabitants in a given country. Moreover, the interest in the coronavirus memes measured on Google Trends returned results for 136 countries. These countries were classified as those where interest in coronavirus memes occurred. The remaining countries where Google Trends did not return any result for search phrases were classified as those in which interest in coronavirus memes did not occur (or was not detectable). We compared these two sets of countries by relating the occurrence of interest in countries to the total number of deaths and cases per one million inhabitants in a given country. Countries where Google Trends detected interest in coronavirus memes were coded as 1 and countries where Google Trends did not detect interest in coronavirus memes were coded as 0.

A search in Google Trends using the terms “covid 19” and “covid” resulted in similar findings; however these terms were much less popular and present in only 20 and 50 countries, respectively. Therefore, we present most of the analyses of these strings in the Supplementary Material.

### 2.2. Comparing the Content of Coronavirus Memes and Random Memes

We selected 200 random coronavirus memes (out of 749) and 200 random non-coronavirus memes (out of 839) found via a Google search using image filtering as described in the second paragraph of Section 2.1. The sample size was determined following the recommendation of Møller and Jennions [60]. According to these authors detecting low and significant correlations would require sample of about 200. Moreover, we choose this sample size because meme content could be classified into about 40 items (see Results) and image content analyses was based on chi-square distribution we wanted to keep expected frequencies equaling at least five.

The classification of relevant images as memes was visually performed. A graphic file was classified as a meme if it contained a recognizable image and text (excluding copyright watermarks). Some memes contained no text and were excluded from analysis (we found only four memes, not related to coronavirus that contained no text). Then, these memes were scrutinized by writing down the text present in the meme and classifying the meme image content. The latter was classified as containing (a) human object, (b) non-human object (e.g., animal), or (c) both. After this, we determined the detailed content of images (e.g., a human object could be a movie actor, scientist, painter, celebrity, ordinary person, etc.; a non-human object could be a cat, dog, bottle, jar, vegetable, sandwich, etc.). There was no specific hypothesis with these categories, and the purpose of this study was to compare the content of coronavirus and non-coronavirus memes.

Apart from the analysis of meme content, we prepared an internet questionnaire to compare the funniness [61] of coronavirus and non-coronavirus memes. Three sets of questionnaires were prepared. Each contained 10 coronavirus memes (randomly chosen from first 100 found via Google search [see: Section 2.1]), 10 non-coronavirus memes, and 10 photos that were not memes (a tree, face, dog, cat, car, bottle, medicine, towel, house, street). Images that were not memes constituted a group that was used to identify potentially spuriously answering participants (see below). The order of the images was randomly shuffled each time. The memes and images were then scored by participants on a five-point Likert-type scale [62,63]: 1 = not funny at all, 2 = somewhat funny, 3 = funny, 4 = very funny, 5 = extremely funny. In the questionnaire, we also asked for the following data: Age of the participant (in years), gender (female, male, prefer not to say), and country of residence. Moreover, in the last question, the participants could (optionally) leave comments. We investigated the raw data to minimalize effects of fake or dishonest respondents (e.g., giving always the same score) but we did not find evidence of spurious answers. These sets of questionnaires were available in the link: 1. https://tinyurl.com/yczetymo 2. https://tinyurl.com/yatnyfxa 3. https://tinyurl.com/y7h9jk6a.

### 2.3. Statistical Data Analyses

All statistical analyses were performed using R version 4.0.3 [64].

#### 2.3.1. Testing Associations among Variables

Associations among the Google Trends interest in coronavirus memes (three search strings: “coronavirus meme”, “covid meme”, “covid 19 meme”), number of web pages containing coronavirus memes, number of webpages with memes (not coronavirus memes), the number of COVID-19 cases per week, and number of deaths from COVID-19 per week were tested by correlation analysis and visualized in ‘ggplot2′ v. 3.3.5 [65] and ‘reshape2′ v. 1.4.4 [66] packages in R. A linear model was used to test if the relative interest in coronavirus memes (measured by the Google Trends index for strings: “coronavirus meme”, “covid meme”, “covid 19 meme”) in different countries was associated with the total number of deaths due to COVID-19 per one million inhabitants of a given country and the total number of cases per one million inhabitants (Figure 1). We also included the number of internet users in a given country as a covariate to control for possible differences in access to the Internet. All variables were log_10_ transformed to homogenize variations and remove the effects of detached observations [67]. The skewness before and after the transformation for the total number of deaths due to COVID-19 per one million inhabitants was 3.186 and −0.463, respectively. The skewness for the number of COVID-19 cases per one million inhabitants was 3.471 before the transformation and −0.493 after. The skewness for the total number of internet users was 8.334 before the transformation and −0.489 after. The skewness for interest in coronavirus memes (Google Trends index for string “coronavirus meme”) was 1.640 before the transformation and −0.741 after. Goodness of fit was measured by *R*^2^, the proportion of variation in the dependent variable explained by the covariates. The assumptions of the linear models were tested with the “performance” v. 0.7.3 [68] and by a global test [69] implemented in “gvlma” v. 1.0.0.3 [70] R packages.

The general linear model (GLM) with binomial error variance and logit link-function was used to test the association between the probability of occurrence of interest in coronavirus memes (measured in Google Trends separately for strings: “coronavirus meme”, “covid meme”, “covid 19 meme”) in different countries and the total number of deaths (log_10_ transformed) due to COVID-19 per one million inhabitants of a given country and the total number of cases (log_10_ transformed) per one million inhabitants (Figure 1). In each model, the number of internet users in a given country (log_10_ transformed) was used as a covariate to control for possible differences in access to the Internet among countries. We used the package “rcompanion” v. 2.4.1 [71] to assess the explained deviance (McFadden pseudo-*R*^2^) in GLMs with binomial error variance. This is one of the most commonly used and straightforward proxies for explained variance in GLMs with binary response variables [72]. The assumptions of the GLMs were tested with the “performance” v. 0.7.3 [68].

#### 2.3.2. Meme Image Content Analysis

Based on the classification performed, we tested if the frequencies of objects differed between coronavirus and non-coronavirus memes (Figure 1). First, we used the chi-square test if the frequency of human and non-human objects differed between coronavirus and non-coronavirus memes (we removed objects classified as both because of low frequency). Second, we calculated the bias-corrected Cramer’s *V* coefficient [73] to test whether the frequency of different objects differed between the two types of memes within human and non-human classes. Cramer’s *V* varies from 0 to 1 with values above 0.25, indicating a strong association between two categorical variables [74]. To calculate Cramer’s *V*, we used ‘sjstats’ R v. 0.18.1 package [75].

#### 2.3.3. Text Analysis and Sentiment Analysis

We performed text mining in ‘tm’ v. 0.7.8 [76,77] and “tidytext” v. 0.3.1 [78] packages in R. Analysis was based on individual words and we applied tidy data rules, for example, words present in memes were converted into singular forms, words such as stopwords, conjunctions, prepositions, determiners, and interjections were removed, numbers were converted into words, and abbreviations were written as full words (for more information about tidy data rules see: [78]). We calculated the frequency of words in coronavirus and non-coronavirus memes. We then used bias-corrected Cramer’s *V* coefficient [73] to test whether the frequencies of particular words were similar between coronavirus and non-coronavirus memes (Figure 1).

Sentiment analysis is the process of extracting an author’s feelings from a written text [78]. In the most basic sense, this means categorizing words as either positive or negative. We used the Bing lexicon [79], available via the ‘get_sentiments ()’ function, to classify words as negative or positive. Then, we used the chi-square test to compare the proportion of positive and negative words between the two types of memes. Visualizations were performed in ‘wordcloud’ v. 2.6 [80] and ‘ggplot2′ v. 3.3.5 [65] R packages.

#### 2.3.4. Funniness of Memes

We used the cumulative link mixed model (CLMM) to compare the funniness (an ordered categorical response variable) of coronavirus memes (coded in the analysis as 1) with non-coronavirus memes (coded as 2) and a sample of images (non-memes; coded as 3). We included age (scaled continuous variable in years), gender (factor variable with women coded as 1, men coded as 2), and interaction between them because perceived humor, including COVID-19 humor, differs between genders [28,29,81] and changes with age [29,82,83]. We included two random factors: Questionnaire identity and person identity nested within the questionnaire’s identity. Two such models were built, for the entire dataset and for the subset that included only Polish participants (the most frequent respondents), respectively. We used the ‘clmm ()’ function from the ‘ordinal’ package v. 2019.12–10 [84] in R. We used the package ‘rcompanion’ v. 2.4.1 [71] to assess the explained deviance (McFadden pseudo-*R*^2^) in CLMM and ‘ggeffects’ v. 1.1.0 [85] to visualize estimates of the models.

## 3. Results

### 3.1. Availability of Coronavirus Memes and Their Popularity

**Figure 2:**
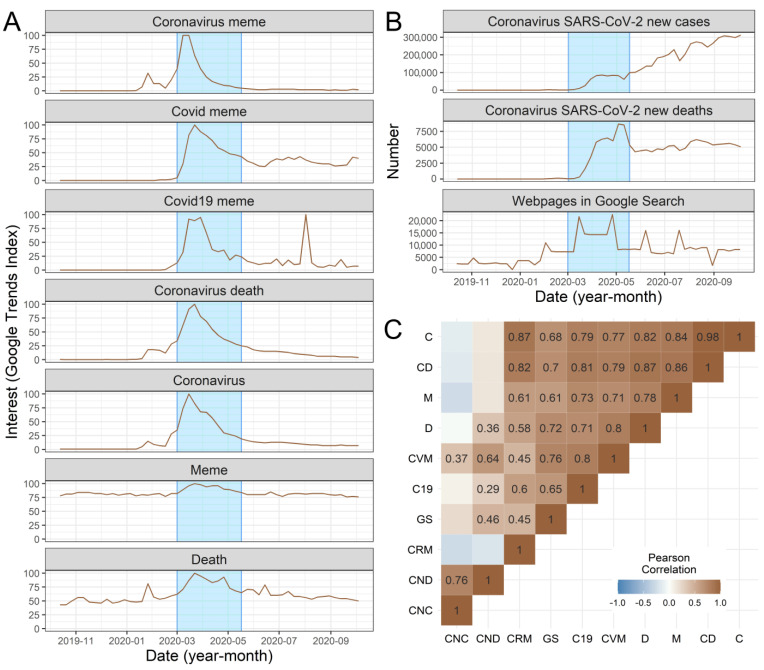
The temporal trends in the selected search strings, coronavirus data, and correlations among analyzed variables. (**A**) The temporal (October 2019–October 2020) trend in the interest (measured as a Google Trends index) in seven different search strings: “coronavirus meme”, “covid meme”, “covid 19 meme”, “coronavirus death”, “coronavirus”, “meme”, “death”. The blue rectangles indicate the period of a spring 2020 lockdown. (**B**) The temporal changes in real numbers of SARS-CoV-2 coronavirus new cases and deaths, and web pages in Google Search containing the phrase: “(coronavirus OR covid 19 OR covid) meme”. (**C**) Correlations among the search strings, real coronavirus data and number of webpages. Only statistically significant associations are shown as numbers. Explanation of codes: CRM–“coronavirus meme”, CVM–“covid meme”, C19–“covid 19 meme”, C–“coronavirus”, CD–“coronavirus deaths”, CNC–coronavirus SAR-CoV-2 new cases, CND–coronavirus SAR-CoV-2 new deaths, D–“death”, M–“memes”, GS–number of web pages in Google search containing the phrase “(coronavirus OR covid 19 OR covid) meme”.

From a Google search, we retrieved 2,150,000 webpages matching the string: “(coronavirus OR covid 19 OR covid) meme”. The relative interest in coronavirus memes (Google Trends index) was highest during the spring lockdown (Figure 2). The temporal variability in the interest in string “coronavirus meme” also strongly positively correlated with the interest in phrases: “coronavirus” (*r* = 0.871, *t* = 12.538, *df* = 50, *p* < 0.001), “coronavirus deaths” (*r* = 0.820, *t* = 10.137, *df* = 50, *p* < 0.001), but only moderately correlated with the interest in simple word: “death” (*r* = 0.583, *t* = 5.077, *df* = 50, *p* < 0.001; Figure 2). The temporal variability in the interest in string “coronavirus meme” was not correlated with the temporal changes in the real numbers of new COVID-19 cases (*r* = −0.272, *t* = −1.997, *df* = 50, *p* = 0.051) or with temporal changes in the number of deaths due to COVID-19 (*r* = −0.196, *t* = −1.411, *df* = 50, *p* = 0.164; Figure 2). Similar results were found for the interest in string “covid 19 meme” (Figure 2). However, the temporal variability in the interest in string “covid meme” was positively correlated with the temporal changes in real numbers of new COVID-19 cases (*r* = 0.369, *t* = 2.804, *df* = 50, *p* = 0.007) and with temporal changes in the number of deaths due to COVID-19 (*r* = 0.640, *t* = 5.901, *df* = 50, *p* < 0.001; Figure 2).

The changes in the number of webpages containing the phrase “(coronavirus OR covid 19 OR covid) meme” were moderately positively correlated with the relative interest in Go ogle Trends for “coronavirus meme” (*r* = 0.446, *t* = 3.523, *df* = 50, *p* < 0.001), “covid 19 meme” (*r* = 0.646, *t* = 5.986, *df* = 50, *p* < 0.001), and “covid meme” (*r* = 0.765, *t* = 8.391, *df* = 50, *p* < 0.001), and positively correlated with changes in the number of deaths due to COVID-19 (0.460, *t* = 3.667, *df* = 50, *p* < 0.001), but not with changes in the number of new cases of COVID-19 (*r* = 0.208, *t* = 1.501, *df* = 50, *p* = 0.139; Figure 2).

### 3.2. The Differences in the Interest in Coronavirus Memes among Countries

**Figure 3:**
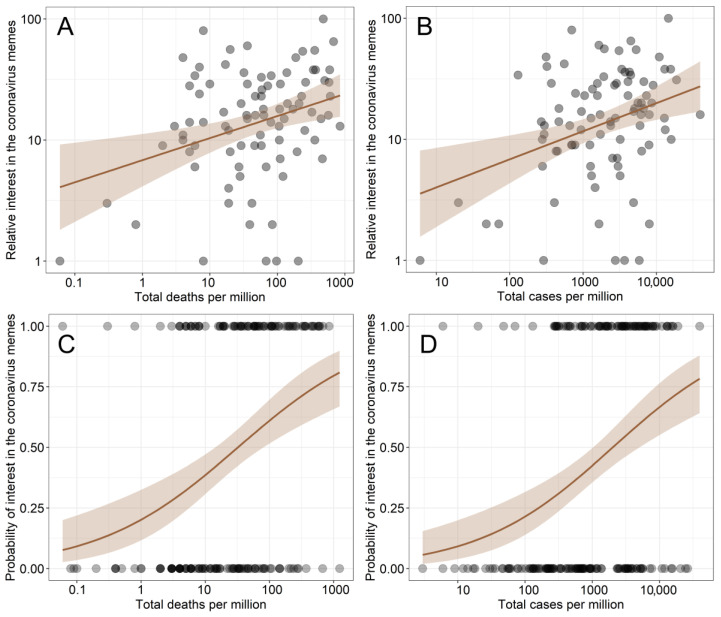
The associations between the interest in coronavirus memes and real coronavirus statistics in different countries. The relative interest (measured as the Google Trends index) in coronavirus memes (search string: “coronavirus meme”) in different countries and its relation to (**A**) total number of deaths due to COVID-19 per one million inhabitants of a given country and (**B**) the total number of cases per one million inhabitants. The probability of occurrence of interest in coronavirus memes in different countries in relation to (**C**) the total number of deaths due to COVID-19 per one million inhabitants of a given country and (**D**) the total number of cases per one million inhabitants. For (**A**,**B**), a linear regression was fitted with a 95% confidence interval, and for (**C**,**D**), a GLM with binomial error distribution was fitted with a 95% confidence interval. The data points are transparent for better visibility of the overlapping values.

The relative interest in coronavirus memes (search string “coronavirus meme”) in different countries was positively associated with the total number of deaths due to COVID-19 per one million inhabitants of a given country (Table 1, Figure 3) and the total number of cases per one million inhabitants (Table 2, Figure 3). The probability of occurrence of interest in coronavirus memes in different countries was also positively associated with the total number of deaths due to COVID-19 per one million inhabitants of a given country (Table 3, Figure 3) and the total number of cases per one million inhabitants (Table 4, Figure 3).

### 3.3. Meme Contents

**Figure 4:**
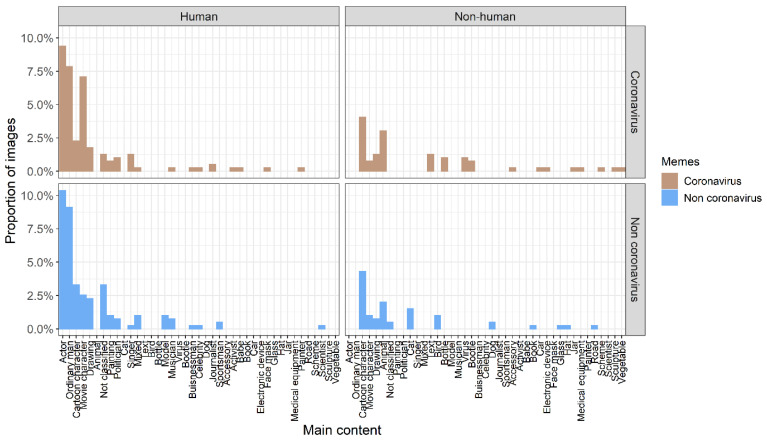
Image content in coronavirus (beige bars) and non-coronavirus memes (blue bars). The image content was divided into human (**left** panel) and non-human (**right** panel) objects.

*Image content.* There was a very similar number of memes containing human objects in coronavirus and non-coronavirus memes (139 versus 146, respectively). The number of memes containing non-human objects in coronavirus and non-coronavirus memes was also similar (60 versus 50, respectively). Only five memes contained both human and non-human objects. Overall, the content of images (proportion of human objects, non-human objects, or both) did not differ between coronavirus and non-coronavirus memes (chi-square test, *ꭕ*^2^ = 2.881, *df* = 2, *p* = 0.237, *N* = 400). When we analyzed visual content in detail, we found that both types of memes were dominated by movie actors or cartoon characters (Figure 4). However, visual content, generally, was similar between coronavirus and non-coronavirus memes for both human objects (Cramer’s *V* = 0.821, 95% CI = 0.742–0.958; Figure 4) and non-human objects (Cramer’s *V* = 0.775, 95% CI = 0.641–0.932; Figure 4).

**Figure 5:**
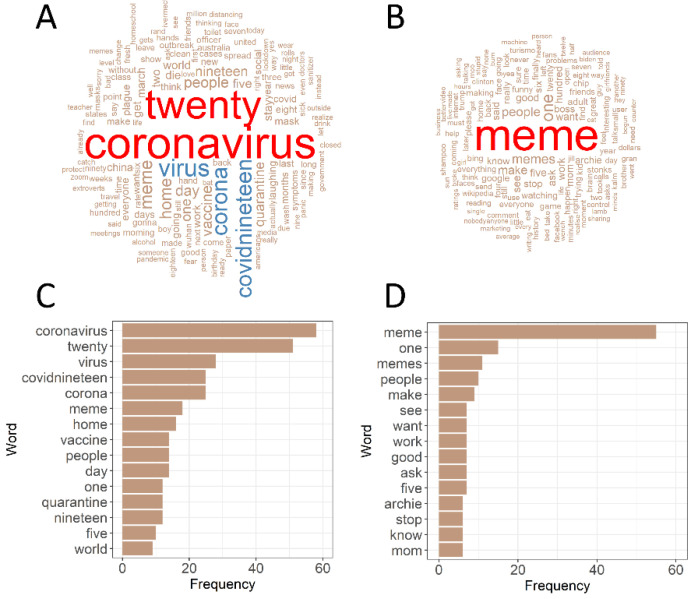
Text mining analysis in coronavirus (**left** panel: (**A**,**C**)) and non-coronavirus memes (**right** panel: (**B**,**D**)). The size of words in the word clouds was scaled by frequency. The 15 most common words are presented in the lower panel.

*Text mining.* Coronavirus memes contained 1,025 words and non-coronavirus memes contained 841 words. The dominant words in coronavirus memes were related to the pandemic (Figure 5), while non-coronavirus words were focused on memes themselves (Figure 5). There was a moderate association between word frequencies in coronavirus and non-coronavirus memes (analysis for all words, Cramer’s *V* = 0.492, 95% CI = 0.390–0.622). Analysis repeated on 30 the most common words found in both types of memes also showed considerable similarity (Cramer’s *V* = 0.566, 95% CI = 0.482–0.729).

*Sentiment analysis.* In coronavirus memes, 125 words (12%) were identified as emotional. Forty-six words were positive and 79 were negative. In non-coronavirus memes, 101 words (12%) were emotional. Forty-two patients were positive and 56 were negative. The differences in the frequency of emotional words (chi-square test *ꭕ*^2^ = 0.003, *df* = 1, *N* = 1866, *p* = 0.959), as well as the proportion of positive versus negative words (chi-square test *ꭕ*^2^ = 0.609, *df* = 1, *N* = 223, *p* = 0.435), were statistically non-significant between coronavirus memes and non-coronavirus memes.

**Figure 6:**
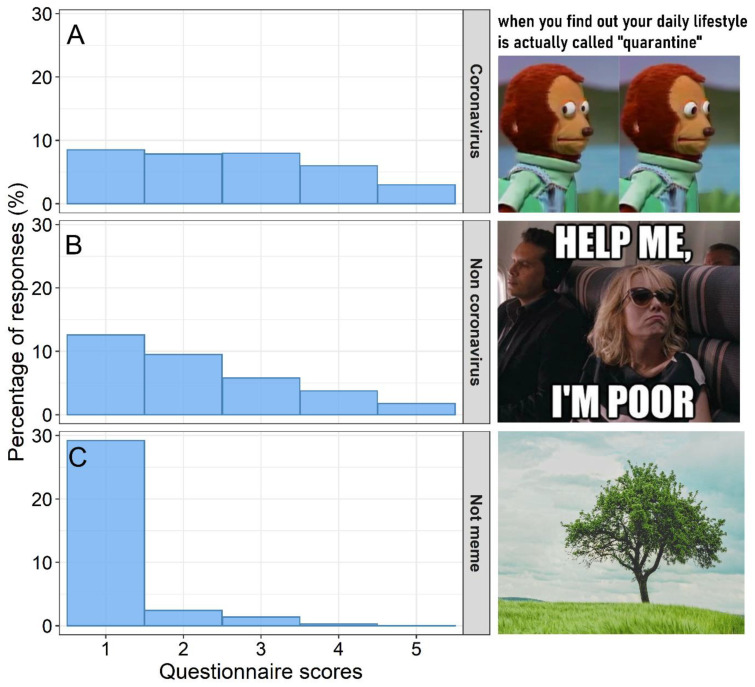
Histograms of funniness scores and exemplary memes and non-memes used in the questionnaire. Frequency distribution (%) of different funniness scores (*x*-axis) for (**A**) coronavirus memes, (**B**) non-coronavirus memes, and (**C**) images not memes (added to check if memes differ from ordinary images). Scores were on the ordinal scale: 1 = not funny at all, 2 = somewhat funny, 3 = funny, 4 = very funny, and 5 = extremely funny.

**Figure 7:**
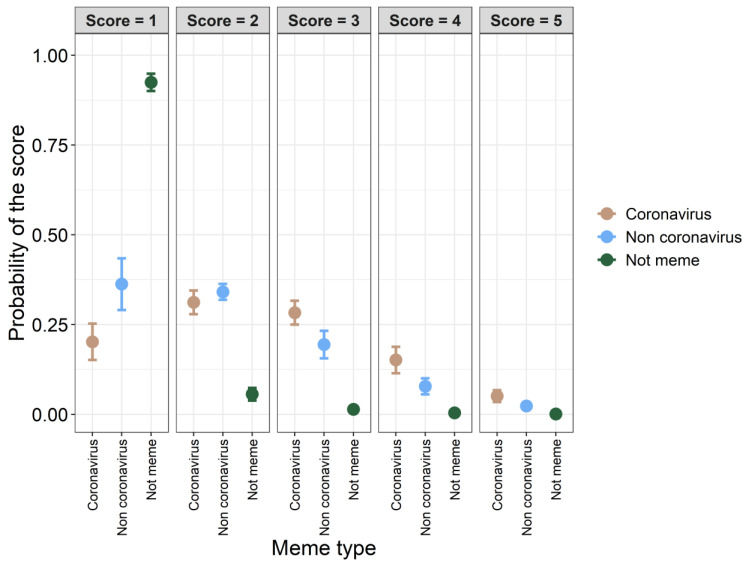
Predicted probability with 95% confidence intervals (whiskers) of the different funniness scores for coronavirus memes, non-coronavirus memes, and sample of images (not memes). The results of the cumulative link mixed model are shown in Table 5. Funniness scores are represented by the gray facet labels. Further explanations are presented in Figure 6.

**Figure 8:**
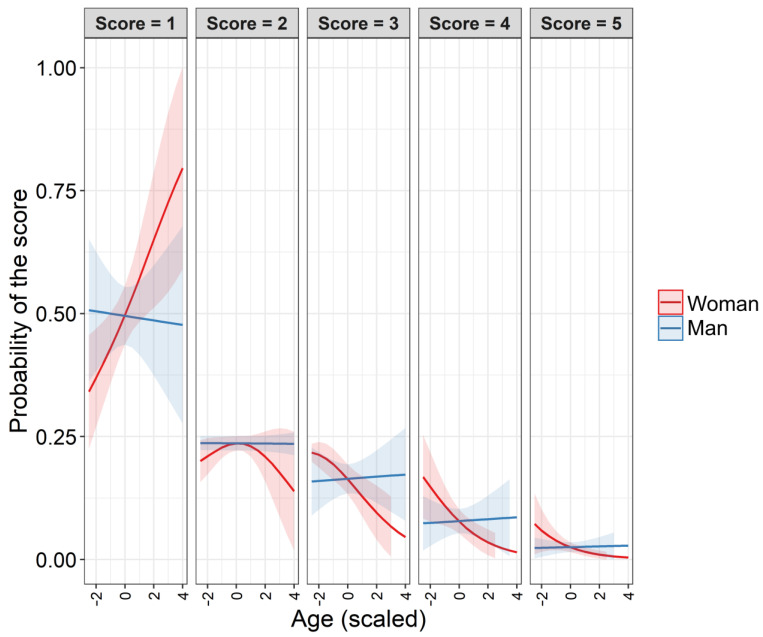
Predicted changes in the probability (lines) with 95% CI (ribbons along lines) of the different funniness scores in two genders across age. The results of the cumulative link mixed model are shown in Table 5. Different funniness scores are represented by the gray facet labels. The *x*-axis is scaled, with zero equaling 35.2 years ± 8.50 SD. Further explanations are presented in Figure 6.

*Funniness of coronavirus memes*. One-hundred-and-thirty-one individuals from 12 countries (Poland, 102 participants; Australia, 10; USA, 4; United Kingdom, 3; Colombia, 3; Philippines, 2; Canada, 1; Chile, 1; France, 1; Germany, 1; India, 1; Sweden, 1; one without exact geographic location) filled out the online questionnaire. Sixty-three participants were women, 65 were men, and three participants did not specify their gender (Table 5). Mean age of participants was 35.2 years ± 8.50 SD, range: 18–65 years). The age-by-gender statistics are presented in Table 5. Coronavirus memes were more frequently valued as humorous than non-coronavirus memes and reference images (non-memes) (Table 6, Figure 6 and Figure 7). The probability that memes received lower scores increased with older participants (Table 6). The effect of gender was not statistically significant (Table 6). However, there was a significant effect from the interaction between gender and age (Table 6, Figure 8). The interaction indicated that older women were more likely to have lower funniness scores than younger women and older men (Figure 8). However, younger women were more likely to have higher funniness scores (3–5) than older women and younger men (Figure 8). Overall, the effect of age on funniness was relatively constant in men (Figure 8). Very similar results were obtained when only Polish participants were analyzed (see Table S9, Figures S15 and S16 in the Supplementary Material).

## 4. Discussion

Social distancing, quarantine, isolation, and fear of death caused by COVID-19 can be overwhelming. Finding strategies to cope with stress is crucial and advised by the World Health Organization to protect people from stress, anxiety, and mental illness [86]. Collective coping theory [87] may provide a basis for managing negative emotions associated with the COVID-19 pandemic [55]. Coping refers to thoughts and behaviors that people use to handle both internal and external stressful situations that are observed through emotional regulation and instrumental problem solving [55,87]. Our research identified a global-scale potential relationship between coping with stress caused by the COVID-19 pandemic and humor. We showed that people dealing with the effects of the pandemic have increased interest in the sociological phenomenon of amusing internet memes and have become exceptionally active in the creation of new memes, especially during the spring 2020 lockdown. This confirms theories and other research showing that turning to humor is one possible strategy for people when they face trauma and stress [88,89], including those associated with COVID-19 [46,55]. However, most previous studies were cohort-based or performed in a single country. The novelty of our research lies in the revealing global pattern of interest in coronavirus memes and explaining differences among countries using coronavirus statistics.

We found that interest in coronavirus memes was positively associated with a higher number of COVID-19 deaths and cases at the country level. The pandemic had different dynamics and trajectories in different countries [90]. In addition, countries differ in culture and policy; thus, people may differentially perceive risk from COVID-19 [91]. Despite these, the correlation between the prevalence of COVID-19 and interest in coronavirus memes was confirmed using different search strings in Google Trends. These interesting findings suggest that the response of societies to COVID-19 has a universal basis.

Why, therefore, are people interested in coronavirus memes? In 2020, COVID-19 was spreading in all countries of the world, which was a novel experience for all living generations, bearing fear, uncertainty, and societal isolation. All people had to face the pandemic, with unknown, potentially dangerous consequences [92]. The unknown generates a fear that is considered a fundamental fear [93]. During the pandemic, many people all over the world were stuck in their homes and transferred much of their lives to the Internet, using it to connect with other people who felt the same way and who were having a similar experience [94,95]. Indeed, internet activity and the use of social media increased during the pandemic [96,97]. Fright, uncertainty, anxiety, or isolation result in the expression of a range of adaptive or defensive behaviors, which are aimed at escaping the source of the danger [98], or building of resilience to it [21]. We believe that interest in coronavirus memes may be an adaptive response that limits the harmful effects of stress associated with the spread of the virus, diverts attention from the chaos of the pandemic, and changes negative emotions into positive ones related to humor. Several studies revealed that interest in memes and humor in general may have increased mental well-being during the pandemic [55,99]. Coronavirus memes also scored highest in funniness compared with other types of pandemic humor [29]. It has been suggested that interactive campaigns (e.g., “post your most humorous covid meme”) may have increased the audience’s involvement and their happiness level during the pandemic [100].

Coronavirus memes may create a realm that distracts from reality [101] and impose different values on the pandemic compared to everyday news. Of course, we cannot exclude the possibility that interest in coronavirus memes was sometimes a side effect of searching for information on the coronavirus [102]. The coronavirus memes may have helped people understand the required behavioral changes and adjustments to preventive routines during the pandemic, for example, wearing a face mask, hand washing, and maintaining social distance. This is unlikely, but memes may also diminish some scientific facts about COVID-19 and, thus, may add to misinformation spread, jeopardizing efforts to stop the pandemic [103]. Anti-vaccination messaging presented in memes may undermine efforts to ensure the widespread uptake of various COVID-19 vaccines [33]. However, the messages encouraging vaccination gained 50% of the total cumulative views, despite being much less abundant than discouraging messages [33].

Although anxiety is a natural adaptive reaction, it can become pathological and interfere with the ability to cope successfully with various stressful events [92,98]. A century ago, during the deadly Spanish flu pandemic, newspapers published some cartoons poking fun at the deadly influenza [104,105]. Cartoons were also published in newspapers in the pre-Internet era during other disasters, such as the sinking of the Titanic [106] or during world wars (e.g., [107]), and they can be treated as prototypes of internet memes and were mentioned as traditional memes by Dawkins [38]. Therefore, comic meme-like constructs were already in use over 100 years ago in a similarly stressful time. The function of these humorous instances was to help in coping with these disastrous conditions by addressing fear while convincing oneself that the danger is under control [108,109]. In addition, the function of this humor could be more complex; for example, humoristic content in newspapers on the sinking of the Titanic symbolically depicted the epic failure of modernity rather than a mere tragic disaster [106]. In general, the use of humor reflects positivity in reframing, as well as active strategies for coping and planning [110], and its duration is tied to its function [111]. One of the responses to the anxiety and uncertainty experienced during the coronavirus pandemic may be people becoming immune to an excess of bad news due to the habituation phenomenon [112]. People in the spring 2020 lockdown experienced difficult emotions about the coronavirus pandemic, such as feelings about becoming sick, government restrictions, or economic slumps [92]. The newspapers on the front page, radio, TV, and social media were filled with stories about the coronavirus pandemic and the latest frightening death toll statistics [113]. The inflow of data on the number of infected people does not make the same impression today as it did during the first lockdown. Despite the spread of coronavirus across the world, people have become fatigued and used to life in the changing circumstances of the global pandemic [114,115]. This could also be seen in the data from Google Trends, where interest in coronavirus memes and coronavirus itself dropped after the spring 2020 lockdown. This may also have positive consequences. It has long been recognized that alongside negative responses to trauma, there can also be positive changes [116,117]. In particular, some traumatized populations not only demonstrate resilience, but also report post-traumatic growth in response to extreme events [117,118]. Survivors value their sense of self-worth or their changed life trajectory following trauma [119,120]. It is not merely the restoration of a person’s pre-trauma state of functioning, but a positive change in previous ways of thinking, indicative of a reorientation of values or priorities in the wake of trauma [117,120,121]. Thus, strategies mitigating the pandemic may rely on the maintenance of positive emotions (e.g., positive reappraisal, problem-focused coping, infusing ordinary events with positive meaning) that buffer against stress [23] and depressed mood [122]. These strategies would help individuals emerge from crises with new coping skills, enhancing psychological well-being [121]. These problems should be studied thoroughly in further research on combating pandemics (e.g., [123]).

Internet viral memes spread through replication on social networks, have a certain longevity, and mutate over time [39,124]. Our results indicate that coronavirus memes “behave” as typical memes. The memes in our study had short durations, and the highest intensity of creation and interest occurred during the relatively short period of the spring 2020 lockdown.

Interest in a particular meme type rarely extends for a longer time. One such example is the phenomenon of amusing internet memes with the proboscis monkey *Nasalis larvatus,* whose popularity lasted for a few years [41]. One possible explanation is that the COVID-19 pandemic was a novel phenomenon during the spring and then societies habituated to the presence of the virus and elevated levels of fear [114,115]. In addition, the diminished use of memes after the spring 2020 lockdown could be that people fully understood the harmful potential of the pandemic and changed their mindset to a more serious one.

We found that coronavirus memes and non-coronavirus memes differed in text content, despite feelings associated with words in the text being similar between the two types of memes. This difference in word content might be responsible for the higher funniness scores of coronavirus memes compared to non-coronavirus memes. Moreover, the distribution of scores given by questionnaire participants was right-skewed. The skewed distribution with predominance of low-rated memes is well known phenomenon because only few memes become viral [125] and are perceived as being very funny [126]. We used sample of randomly chosen memes that probably contained both viral and less funny memes and future studies should identify viral coronavirus memes and compare them with viral non-coronavirus ones. However, researchers are still trying to predict (with sophisticated analytical methods) which memes will become viral, with moderate success [46,51,52]. The intended meaning of the meme is multimodal and needs to integrate text, context, and the current socioeconomic situation [127]. Therefore, it is possible that our simple analysis of meme content might not have detected specific word connections with images or wordplay. Humor is a complex phenomenon, and detecting patterns in the interplay between image content and text may be especially difficult in memes. It is also probable that the pandemic affected participants in a manner that caused them to score coronavirus memes higher. Naturally, the pandemic is a core interest for most people [128], and this may have transferred into interest in and valuing memes. In a broader sense, the slight differences between coronavirus and non-coronavirus memes show that memes evolve via “mutations,” which is a typical feature of a meme [38,101]. These “mutations” are linked with text rather than images. Indeed, both types of memes mostly use photos of actors, cartoon characters, and pets in similar amounts. It is known that movie actors are common subjects in memes [129,130,131]. Our analysis also revealed that memes had higher scores than the reference images (non-memes). This is an expected result that increases trust in the respondents’ answers.

Of course, memes are not the only way to share humorous content. Pandemic humor can be spread simply as oral jokes during direct interpersonal contact, or via traditional media, such as television, newspapers, or live events, such as stand-up comedy shows [95]. Bischetti, Canal and Bambini [29] demonstrated that among analyzed humorous constructs, memes were scored as the funniest, although some were also assessed as aversive.

We found that scores of the coronavirus memes were dependent on the interaction between the age and gender of the participants. Older women were more likely to give lower scores and less likely to give higher scores on the questionnaire than younger women. Meanwhile, the probability of giving a specific score was independent of age in men. This result is similar to other findings [28,82]. Bischetti, Canal, and Bambini [29] found that with increasing age and in women, COVID-19 humor was judged as more aversive, although among the considered types of humor, memes scored the highest. Older adults have greater difficulty with humor comprehension due to age-related cognitive decline compared to young adults [28]. In addition, the older population does not seem to enjoy aggressive types of humor as much as the younger population, and the elderly are especially sensitive to jokes referring to old age [81,132]. Meanwhile, older adults may be better at emotional regulation than their younger counterparts, react to a crisis with less anger, and are better able to adapt their coping strategies to changing environments [133,134].

### Study Limitations

Our study relied on data from webpages spread across the Internet. This imposed certain limitations on the data structure and interpretation of the results. The construction of the time-series (number of webpages with coronavirus memes) from Google searches was not perfect. For example, it was not possible to fully account for image repetition. Moreover, webpages with coronavirus memes appeared as early as October 2019. We scrutinized several of these webpages and found that they were built before the pandemic, but contained “suggestion links” to current new memes. Thus, the series used in this study reflected certain properties of the Google proprietary algorithms, which are impossible to fully control, introducing one more arbitrary aspect. We cannot exclude the possibility that memes on other coronaviruses (e.g., MERS-CoV and SARS-CoV) were built before the COVID-19 pandemic and were associated with the search terms. However, the time-series had a logical interpretation: Despite webpages with coronavirus memes being found in October-November 2019, the highest number of webpages was created during the spring 2020 lockdown. These limitations do not apply to Google Trends. Additionally, the webpages found via Google search included some social media e.g., Reddit but not Facebook or Twitter webpages, probably because of privacy setting in these media. This may create a large gap in the meme sharing behavior. However, Google’s search algorithm usually returns the most popular results first, thus perhaps those spread via social media.

Humor research suffers from an optimistic bias, meaning researchers focus on the positive aspects of the phenomenon, ignoring its darker, negative aspects [135]. Very little attention has been paid to humor that somehow fails to achieve its perlocutionary goal, i.e., to elicit amusement [136]. In the online questionnaire, there was no measure for the negative dimension of humor appreciation, namely aversiveness, quantifying the disturbing potential of some forms of COVID-19 humor [29]. We assumed memes to be related to humor and, thus that they should generate positive emotions or, at worst, no emotions. This is a limitation, but we believe that including negative values would only deepen the differences between coronavirus memes and non-coronavirus memes. Category “1” in our questionnaire, meaning “not funny at all” probably encompassed all these negative judgments and was the most frequent in the non-coronavirus memes.

Most of our analyses were correlative. However, studies on a global scale rarely, if at all, are experiments. Our general and generalized linear models explained usually below 40% of variance. This indicates that we did not include some predictors or there is high random variance in the dependent variables. However, in non-experimental studies regression models usually explain less than 20% of variance in dependent variables [60]. Nevertheless, our findings may provide a good basis for experiments using memes.

Our online questionnaire had a geographic bias, as 75% of respondents came from Poland. We intended to include more participants from other countries, however, without success, probably because we distributed the questionnaire using the snowball sampling technique: the survey was shared with a small pool of respondents who participate, and each of those participants shares the link with their network or pool of respondents [137].

All our memes were in English. We decided to use them because searches in Polish resulted in English memes (many memes in English are simply published or shared on Polish websites). This is probably because the text in memes is just a graphic and Google search recognizes the origin of the website, but it does not recognize the content of the meme. We believe this is not a serious problem because over 60% of citizens in Poland (aged 20-50 years) possess at least basic proficiency in English, and Poland is ranked 16th among 112 countries in English proficiency [138]. Moreover, the average scores of comicality were slightly lower in Poland than in other countries, but the difference was not statistically significant. Furthermore, statistical models built separately on all data and for Polish participants gave very similar results. Thus, we believe this bias probably had little impact on the results and inference.

## 5. Conclusions

Our findings suggest that societies responded to the threat linked to the pandemic by searching for ways of releasing stress. Interest in memes may allow not only a way of coping with anxiety, fear, or uncertainty, but also a way of shaping attitudes to the novel coronavirus and participating and developing social networks during the pandemic. The occurrence of positive emotions associated with memes may provide the necessary psychological rest to help buffer against stress, replenish, and restore further coping efforts. Moreover, the dynamics of the number and interest in coronavirus memes have some properties of the real virus disease trajectory. However, the dynamics do not follow the COVID-19 pattern in new cases or deaths.

## Figures and Tables

**Table 1:** Results of the linear model testing the association between the relative interest in coronavirus memes (Google Trends index for search string: “coronavirus meme”) and total number of deaths due to COVID-19 per one million inhabitants and the number of internet users in different countries. Estimates of functions slopes are given with standard errors (SE), 95% confidence intervals (LL–lower confidence intervals, UL–upper confidence intervals) and *t*–test (with residual degree of freedom). Model explained 33% variation in data (adjusted *R*^2^).

Variable	Estimate	*SE*	*LL*	*UL*	*t(87)*	*p*
(Intercept)	2.998	0.390	2.221	3.774	7.676	<0.001
Number of deaths per 1M inhabitants of a country (log_10_ transformed)	0.220	0.051	0.118	0.322	4.301	<0.001
Number of internet users in a country (log_10_ transformed)	−0.319	0.056	−0.430	−0.208	−5.708	<0.001

**Table 2:** Results of the linear model testing the association between the relative interest in coronavirus memes (Google Trends index) and total number of COVID-19 cases per one million inhabitants and percentage of internet users in different countries. Estimates of functions slopes are given with standard errors (SE), 95% confidence intervals (LL–lower confidence intervals, UL–upper confidence intervals) and *t*–test (with residual degree of freedom). Model explained 27% variation in data (adjusted *R*^2^).

Variable	Estimate	*SE*	*LL*	*UL*	*t(86)*	*p*
(Intercept)	5.047	1.018	3.024	7.070	4.959	<0.001
Number of COVID-19 cases per 1M inhabitants of a country (log_10_ transformed)	0.573	0.145	0.285	0.861	3.954	<0.001
Number of internet users in a country (log_10_ transformed)	−0.619	0.132	−0.882	−0.356	−4.675	<0.001

**Table 3:** Results of the GLM testing the association between the occurrence of interest in coronavirus memes (Google Trends index) and total number of deaths due to COVID-19 per one million inhabitants and percentage of internet users in different countries. Odds ratio (OR) with 95% confidence intervals (LL–lower confidence intervals, UL–upper confidence intervals) and Wald z–test (with residual degree of freedom). Model explained 33% variation in data (McFadden pseudo-*R*^2^).

Variable	*OR*	*LL*	*UL*	*z(186)*	*p*
(Intercept)	0.00005	0.0004	<0.0001	−6.695	<0.001
Number of deaths per 1M inhabitants of a country (log_10_ transformed)	3.431	2.142	5.830	4.852	<0.001
Number of internet users in a country (log_10_ transformed)	5.082	3.153	8.873	6.193	<0.001

**Table 4:** Results of the GLM testing the association between the occurrence of interest in coronavirus memes (Google Trends index) and total number of COVID-19 cases per one million inhabitants and percentage of internet users in different countries. Odds ratio (OR) with 95% confidence intervals (LL–lower confidence intervals, UL–upper confidence intervals) and Wald z–test (with residual degree of freedom). Model explained 37% variation in data (McFadden pseudo-*R*^2^).

Variable	*OR*	*LL*	*UL*	*z(209)*	*p*
(Intercept)	<0.0001	<0.0001	0.0000	−7.499	<0.001
Number of COVID-19 cases per 1M inhabitants of a country (log_10_ transformed)	3.623	2.244	6.192	4.999	<0.001
Number of internet users in a country (log_10_ transformed)	2.056	1.689	2.588	6.645	<0.001

**Table 5:** Descriptive statistics for age and gender of the participants that completed the online questionnaire.

Gender	Mean	*SD*	Minimum	Maximum	*N*
Women	34.8	8.44	18	67	63
Men	35.8	8.68	18	61	65
Not specified	32.3	6.81	27	40	3

**Table 6:** Results of the cumulative link mixed model comparing funniness of coronavirus memes with non-coronavirus memes and sample of images (not a meme). The threshold coefficients show the transition chances in the questionnaire score point to the next. The model included effects of age, sex, and interaction between age and sex. Random factors were questionnaire identity and participants identity (nested in questionnaire identity). Odds ratio (OR) with 95% confidence intervals (LL–lower confidence intervals, UL–upper confidence intervals) and Wald z–test (with effective degrees of freedom). Model explained 17% variation in data (McFadden pseudo-*R*^2^).

Variable	*OR*	*LL*	*UL*	*z(11)*	*P*
Threshold coefficients:					
1|2	0.255	0.172	0.378	−6.762	<0.001
2|3	1.066	0.720	1.579	0.320	0.749
3|4	3.965	2.668	5.891	6.818	<0.001
4|5	18.743	12.374	28.391	13.833	<0.001
Fixed effects:					
Meme = Non coronavirus	0.445	0.384	0.516	−10.738	<0.001
Meme = Not meme	0.021	0.016	0.026	−33.059	<0.001
Age	0.631	0.442	0.899	−2.550	0.011
Gender = Man	1.015	0.631	1.633	0.060	0.952
Age × Gender = Man	1.634	1.010	2.644	2.001	0.045

## Data Availability

Data supporting reported results can be found in the Supplementary Materials to this article.

## Supplementary Materials

The following supporting information can be downloaded at: https://www.mdpi.com/article/10.3390/ijerph191912969/s1, File S1: Supplementary analyses and results.docx–Testing model assumptions and additional results. File S2: memes_country_data.xlsx–contains global and country-level data on interest in memes and COVID-19 statistics. File S3: Meme_text_data.xlsx–contains data on the analyzed memes and their the text content. File S4: Results_questionnaire.xlsx–contains results from the online questionnaire. File S5: coronavirus_memes_cleaned.txt–contains cleaned text data in the coronavirus memes. File S6: non_coronavirus_memes_cleaned.txt–contains cleaned text data in the non-coronavirus memes. File S7: All_analyses_memes.R–contains codes to reproduce the results.
