# Supplementary material for: COVID-19 in Memes: The Adaptive Response of Societies to the Pandemic?
Source: Int J Environ Res Public Health. 2022 Oct 10;19(19):12969. doi: 10.3390/ijerph191912969 (PMC9566158; doi:10.3390/ijerph191912969)
Supplement: Supplementary file 1 [file ijerph-19-12969-s001.zip › ijerph-1941197-supplementary/File S1. Supplementary_analyses_and_results.pdf]

Supplementary material

TESTING MODEL ASSUMPTIONS AND ADDITIONAL RESULTS

Search string: **"Coronavirus meme"**

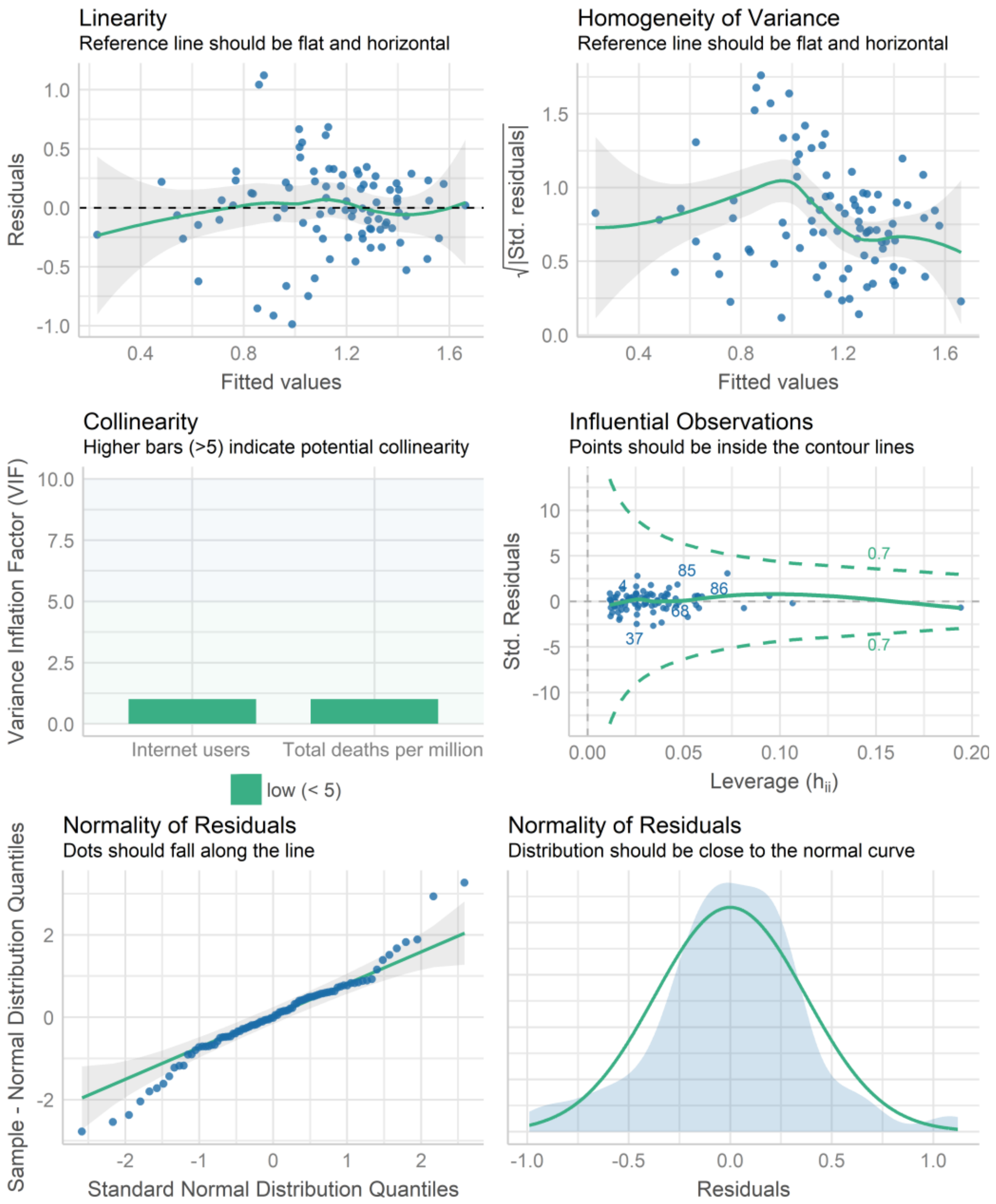

Figure S1. Tests of assumptions of the linear model for association between the relative interest in coronavirus memes (Google Trends index for string: "coronavirus meme") and total number of deaths due to COVID-19 per 1 million of inhabitants, with the number of internet users in a given country as a covariate. Global test revealed no violation of assumptions of linear model.

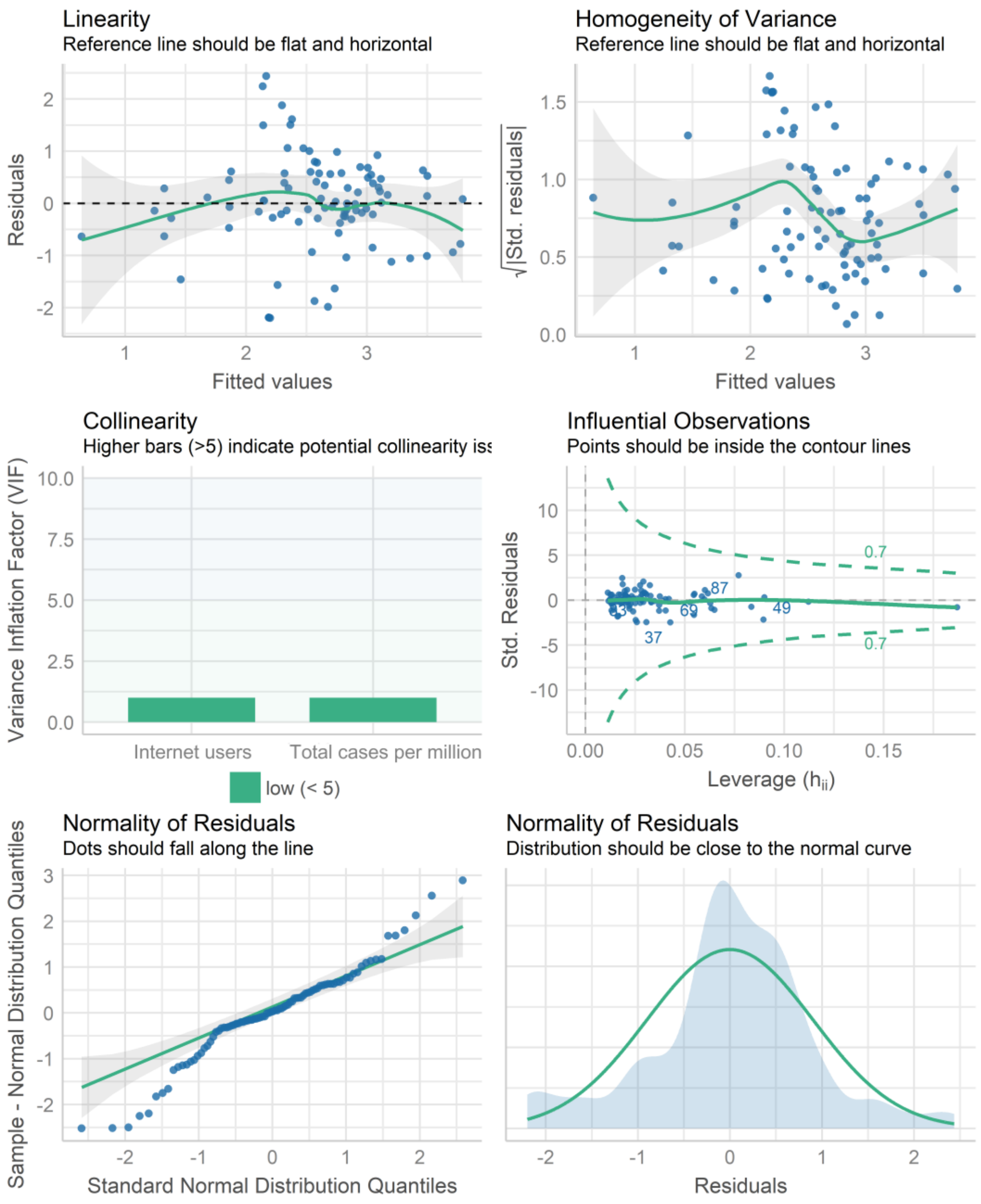

Figure S2. Tests of assumptions of the linear model for association between the relative interest in coronavirus memes (Google Trends index for string: "coronavirus meme") and total number of COVID-19 cases per 1 million of inhabitants, with the number of internet users in a given country as a covariate. Global test revealed no violation of assumptions of linear model.

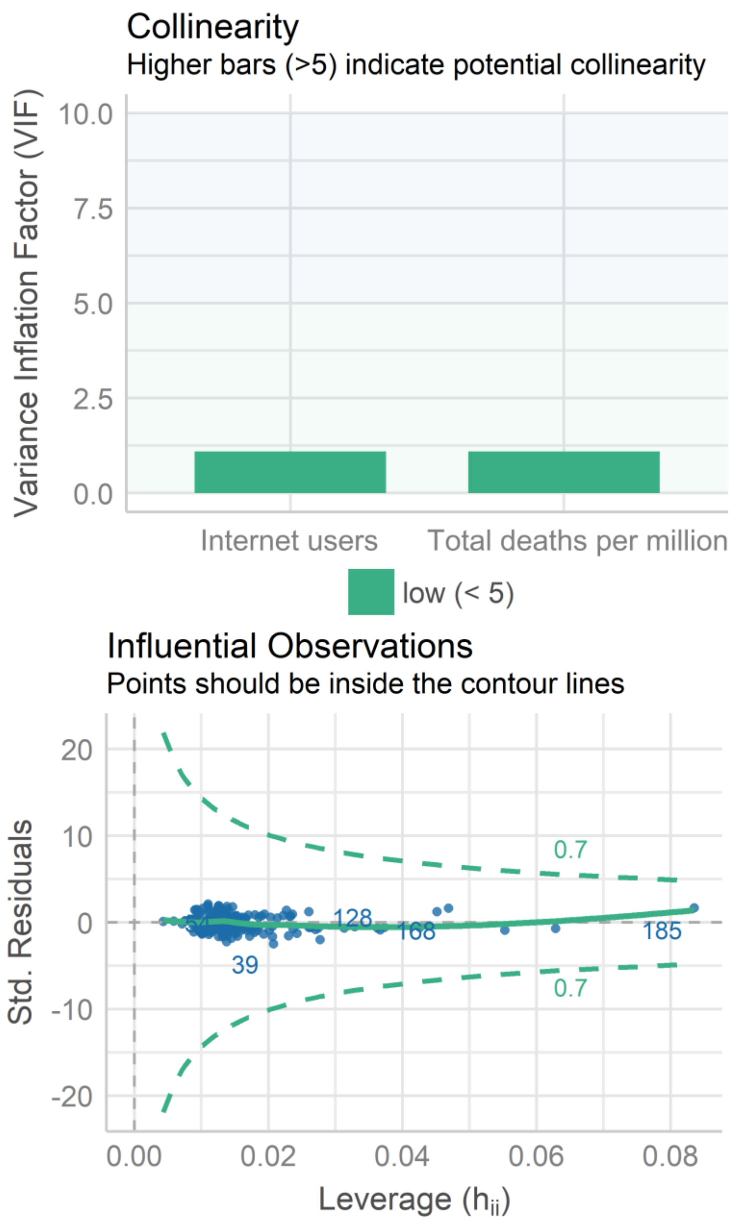

Figure S3. Tests of assumptions of the general linear model with the logit-link function and binomial error distribution, testing association between the occurrence of relative interest in coronavirus memes (Google Trends index for string: "coronavirus meme") and total number of deaths due to COVID-19 per 1 million of inhabitants, with the number of internet users in a given country as a covariate.

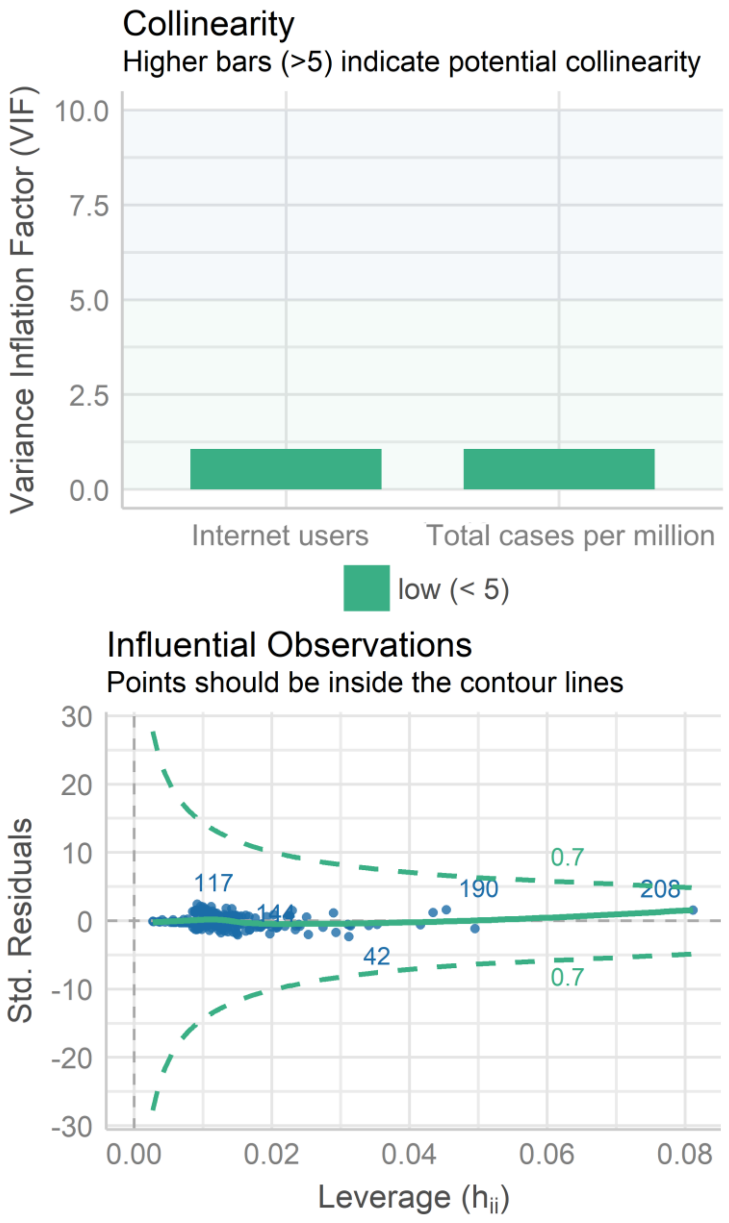

Figure S4. Tests of assumptions of the general linear model with the logit-link function and binomial error distribution, testing association between the occurrence of relative interest in coronavirus memes (Google Trends index for string: “coronavirus meme”) and total number of COVID-19 cases per 1 million of inhabitants, with the number of internet users in a given country as a covariate. Global test revealed no violation of assumptions of linear model.

Search string: **"Covid meme"**

Table S1. Results of the linear model testing the association between the relative interest in coronavirus memes (Google Trends index for search string: "covid meme") and total number of deaths due to COVID-19 per 1 million of inhabitants, with the number of internet users in a given country as a covariate Estimates of functions slopes are given with standard errors (se), 95 % confidence intervals (LL - lower confidence intervals, UL - upper confidence intervals), *t* - test (with residual degree of freedom) and statistical significance (*p*). Model explained 13 % variation in data (adjusted R^2).

**Variable**	**estimate**	***se***	***LL***	***UL***	***t(84)***	***P***
(Intercept)	2.358	0.515	1.334	3.381	4.581	<0.001
Deaths per 1M in a country (log10 transformed)	0.156	0.058	0.041	0.271	2.695	0.009
Number of internet users in a country (log10 transformed)	-0.223	0.073	-0.368	-0.079	-3.069	0.003

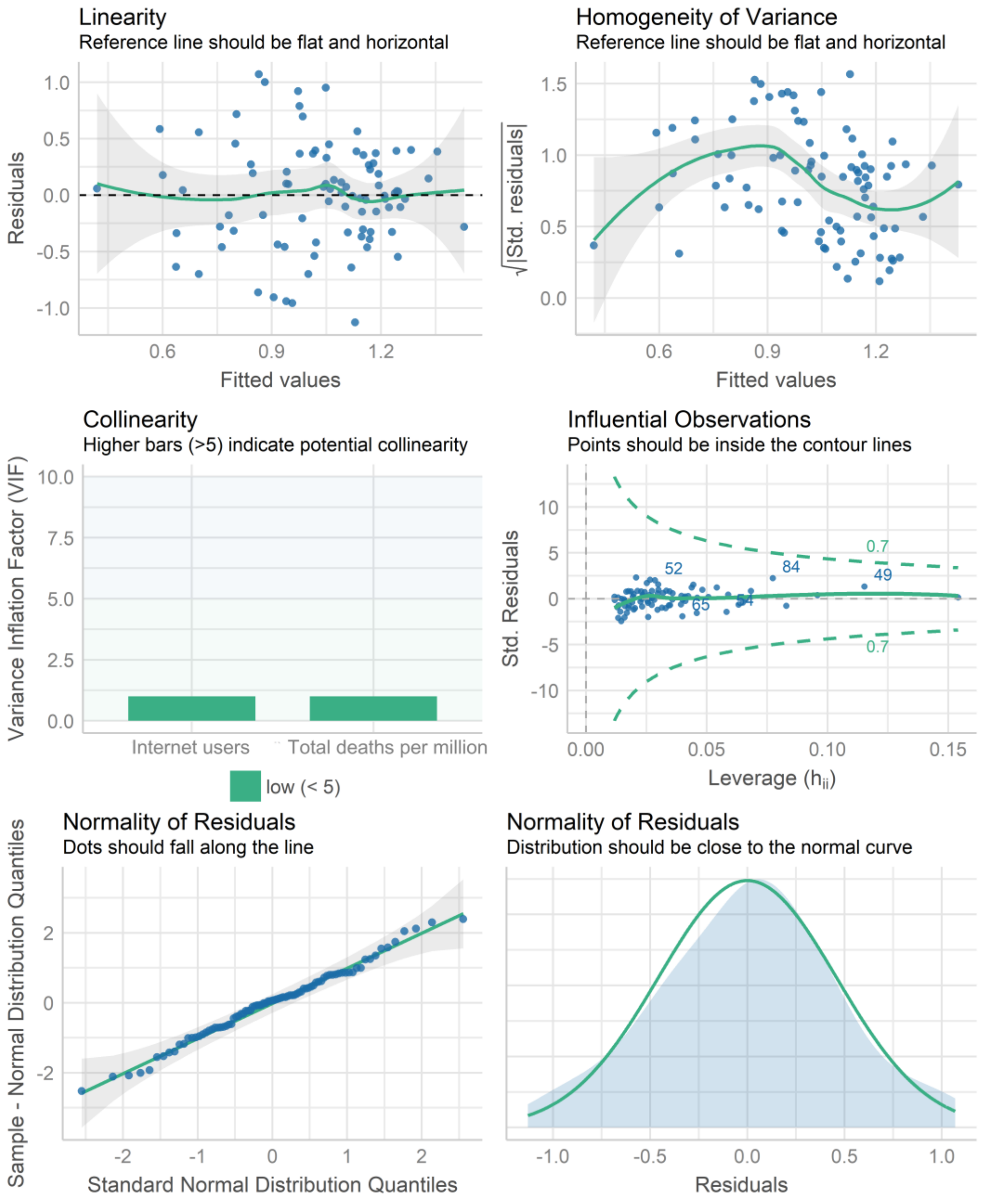

Figure S5. Assumptions of the linear model for testing association between the relative interest in coronavirus memes (Google Trends index for string: "covid meme") and total number of deaths due to Covid-19 per 1 million of inhabitants, with the number of internet users in a given country as a covariate. Global test revealed no violation of assumptions of linear model. Results of the model see: Table S1.

Table S2. Results of the linear model testing the association between the relative interest in coronavirus memes (Google Trends index for search string: "covid meme") and total number of Covid-19 cases per 1 million of inhabitants, with the number of internet users a given country as a covariate. Estimates of functions slopes are given with standard errors (se), 95 % confidence intervals (LL - lower confidence intervals, UL - upper confidence intervals), *t* - test (with residual degree of freedom) and statistical significance (*p*). Model explained 14 % variation in data (adjusted R^2).

**Variable**	**estimate**	***Se***	***LL***	***UL***	***t(84)***	***P***
(Intercept)	1.872	0.560	0.759	2.984	3.345	0.001
Cases per 1M in a country (log10 transformed)	0.191	0.070	0.051	0.331	2.717	0.008
Number of internet users in a country (log transformed)	-0.205	0.072	-0.349	-0.062	-2.840	0.006

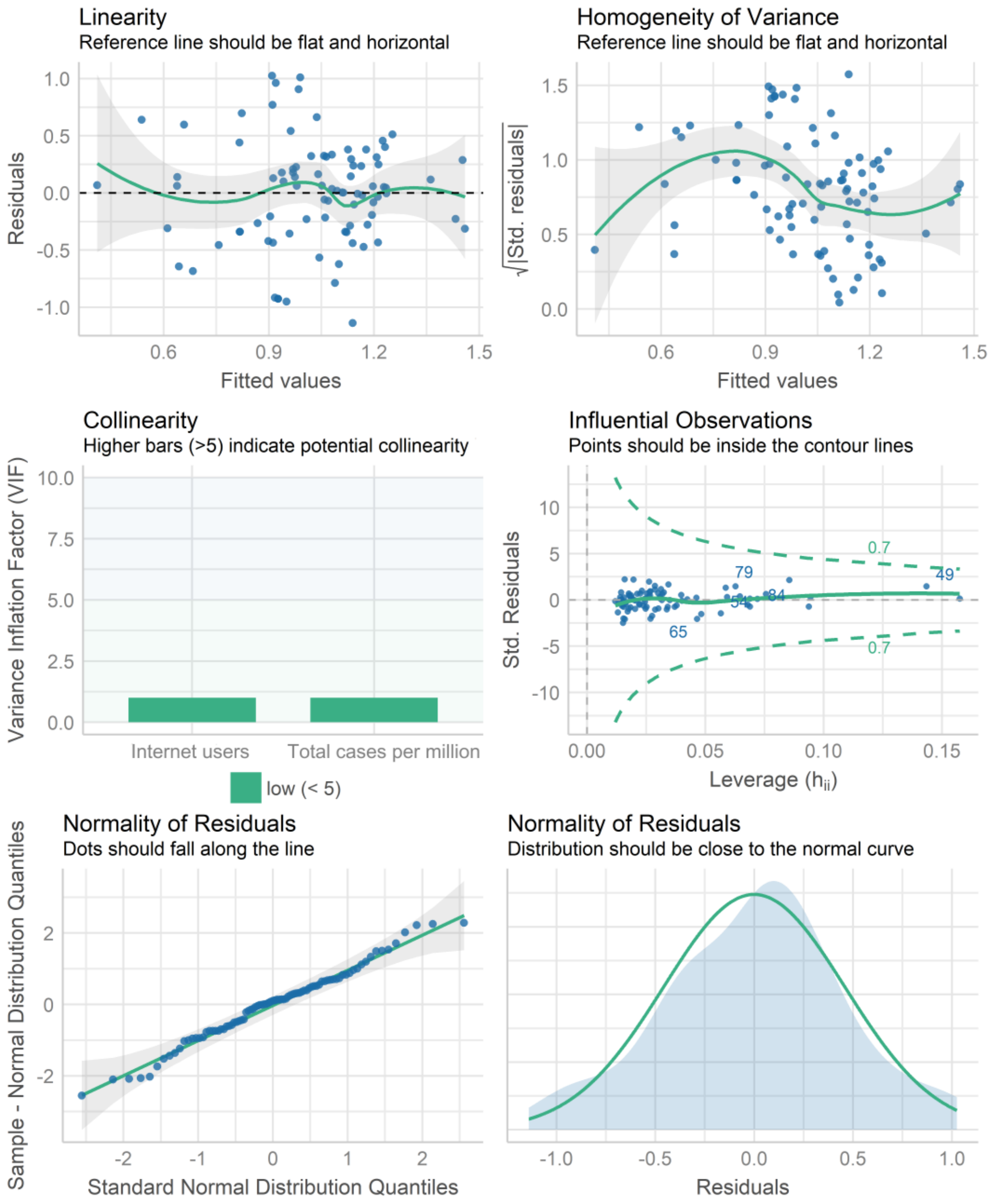

Figure S6. Tests of assumptions of the linear model for association between the relative interest in coronavirus memes (Google Trends index for string: "covid meme") and total number of COVID-19 cases per 1 million of inhabitants, with the number of internet users in a given country as a covariate. Global test revealed no violation of assumptions of linear model. Results of the model see: Table S2.

Table S3. Results of the general linear model testing the association between the occurrence of interest in coronavirus memes (Google Trends index for search string “covid meme”) and total number of deaths due to COVID-19 per 1 million of inhabitants, and number of internet users in a given country as a covariate. Odds ratio (*OR*) with 95 % confidence intervals (LL - lower confidence intervals, UL - upper confidence intervals), Wald *z* - test (with residual degree of freedom) and statistical significance (*p*). Model explained 32 % variation in data (McFadden pseudo-R^2).

**Variable**	***OR***	***LL***	***UL***	***z(186)***	***P***
(Intercept)	<0.001	<0.0001	0.001	-6.664	<0.001
Deaths per 1M in a country (log10 transformed)	2.076	1.355	3.281	3.259	0.001
Number of internet users in a country (log10 transformed)	5.973	3.592	10.897	6.350	<0.001

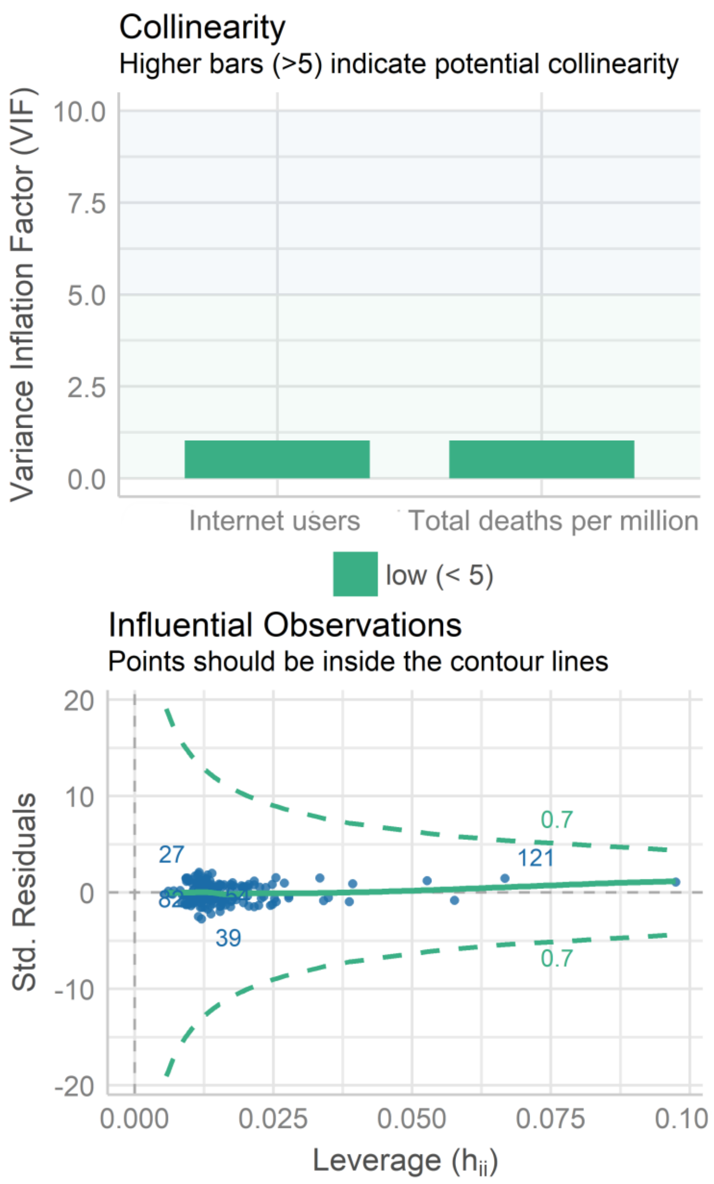

Figure S7. Tests of assumptions of the general linear model with the logit-link function and binomial error distribution, testing association between the occurrence of relative interest in coronavirus memes (Google Trends index for string: "covid meme") and total number of deaths due to COVID-19 per 1 million of inhabitants, with the number of internet users in a given country as a covariate. Results of the model see: Table S3.

Table S4. Results of the general linear model testing the association between the occurrence of interest in coronavirus memes (Google Trends index for search string "covid meme") and total number of COVID-19 cases per 1 million of inhabitants, and number of internet users in a given country as a covariate. Odds ratio (*OR*) with 95 % confidence intervals (LL - lower confidence intervals, UL - upper confidence intervals), Wald *z* - test (with residual degree of freedom) and statistical significance (*p*). Model explained 37 % variation in data (McFadden pseudo-R^2).

**Variable**	***OR***	***LL***	***UL***	***z(209)***	***P***
(Intercept)	<0.001	<0.001	0.000	-7.284	<0.001
Cases per 1M in a country (log10 transformed)	2.346	1.501	3.783	3.625	<0.001
Number of internet users in a country (log10 transformed)	6.676	4.034	12.163	6.782	<0.001

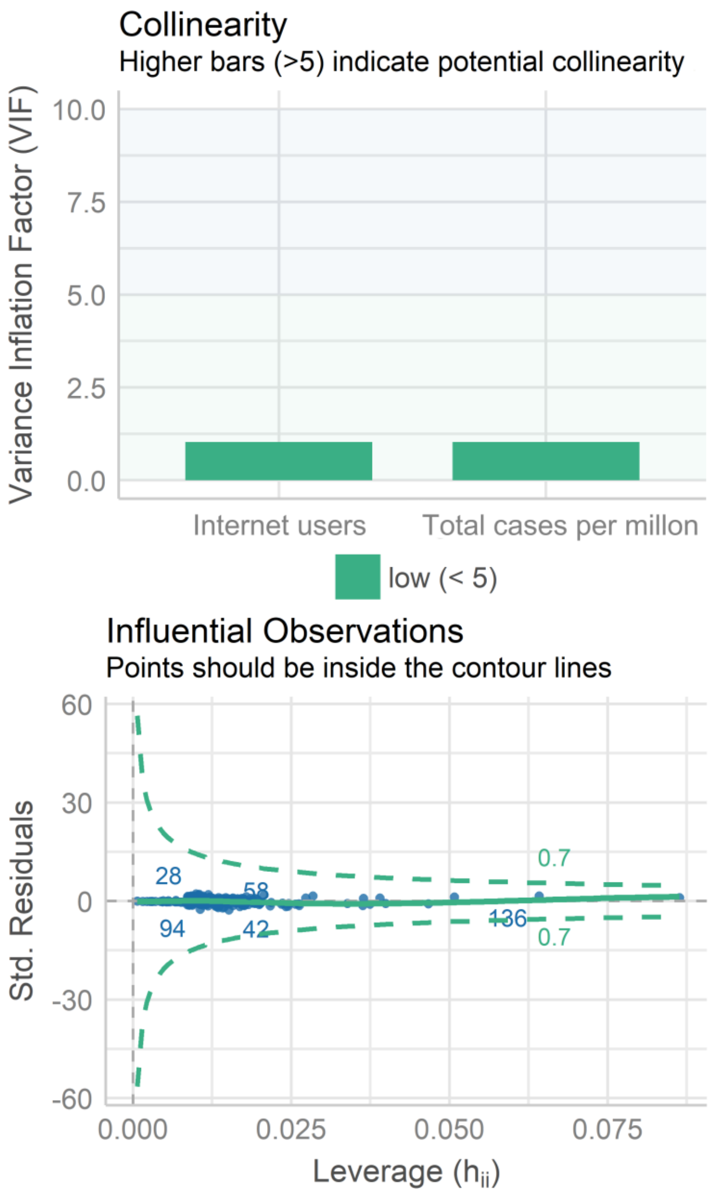

Figure S8. Tests of assumptions of the general linear model with the logit-link function and binomial error distribution, testing association between the occurrence of relative interest in coronavirus memes (Google Trends index for string: "covid meme") and total number of COVID-19 cases per 1 million of inhabitants, with the number of internet users in a given country as a covariate. Results of the model see: Table S4.

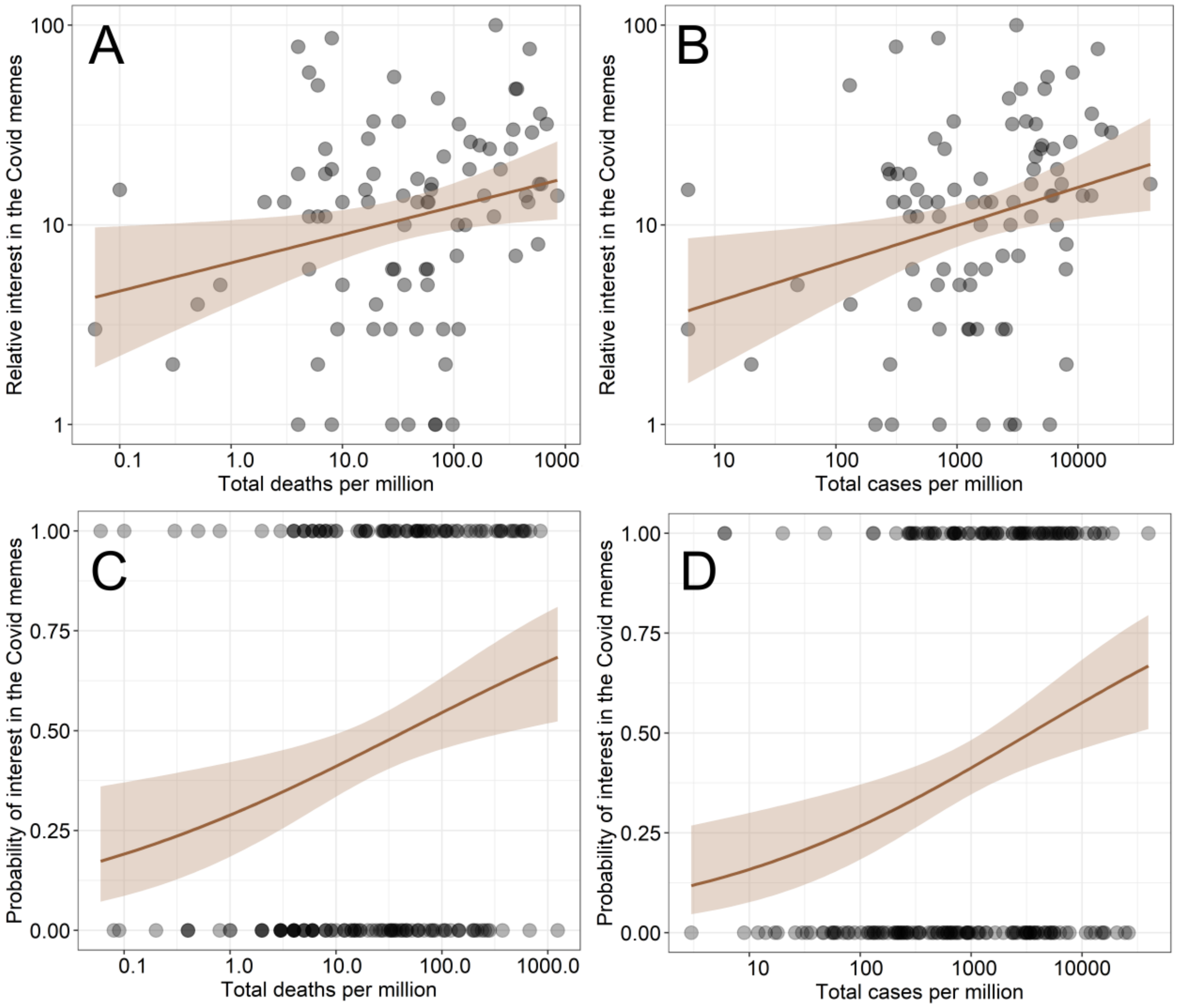

Figure S9. The associations between the interest in coronavirus memes and coronavirus statistics in different countries. The relative interest (measured as the Google Trends index for search string "covid meme") in coronavirus memes in different countries and its relation to (A) total number of deaths due to COVID-19 per 1 million inhabitants of a given country and (B) the total number of COVID-19 cases per 1 million inhabitants. The probability of occurrence of interest in coronavirus memes in different countries in relation to (C) the total number of deaths due to COVID-19 per 1 million inhabitants of a given country and (D) the total number of cases per 1 million inhabitants. For A-B a linear regression is fitted with 95% confidence interval, for C-D a logistic regression is fitted with 95% confidence interval. Data points are transparent for better visibility of overlapping values.

Search string: **“Covid19 meme”**

Table S5. Results of the linear model testing the association between the relative interest in coronavirus memes (Google Trends index for search string: “covid19 meme”) and total number of deaths due to COVID-19 per 1 million of inhabitants, with the number of internet users in a given country as a covariate Estimates of functions slopes are given with standard errors (se), 95 % confidence intervals (LL - lower confidence intervals, UL - upper confidence intervals), *t* - test (with residual degree of freedom) and statistical significance (*p*). Model explained 26 % variation in data (adjusted R^2).

**Variable**	**estimate**	***se***	***LL***	***UL***	***t(16)***	***P***
(Intercept)	3.554	1.152	1.111	5.996	3.085	0.005
Deaths per 1M in a country (log10 transformed)	-0.180	0.097	-0.407	0.048	-1.677	0.113
Number of internet users in a country (log10 transformed)	-0.268	0.156	-0.599	0.063	-1.716	0.105

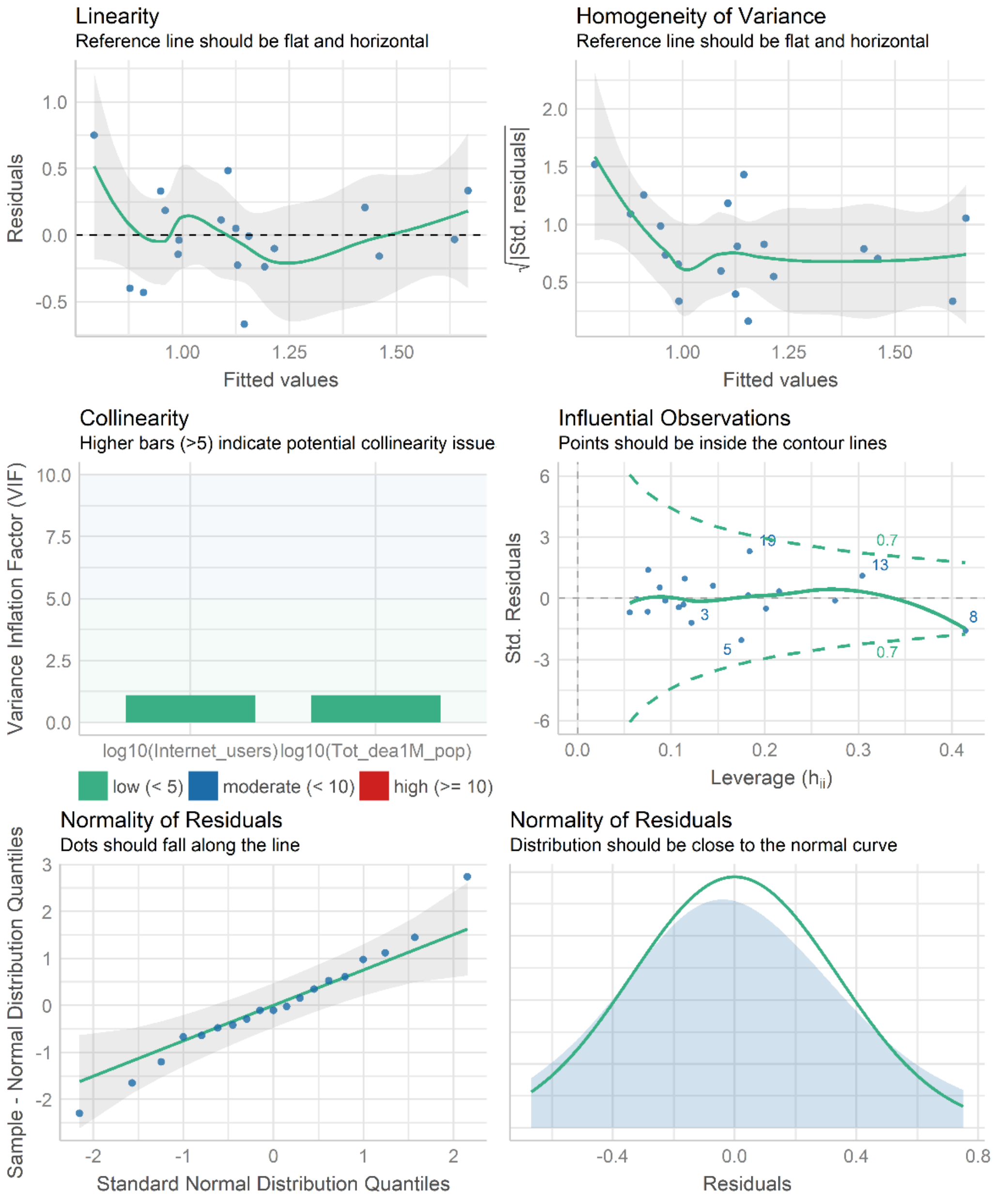

Figure S10. Assumptions of the linear model for testing association between the relative interest in coronavirus memes (Google Trends index for string: “covid19 meme”) and total number of deaths due to Covid-19 per 1 million of inhabitants, with the number of internet users in a given country as a covariate. Global test revealed no violation of assumptions of linear model. Results of the model see: Table S5.

Table S6. Results of the linear model testing the association between the relative interest in coronavirus memes (Google Trends index for search string: "covid19 meme") and total number of Covid-19 cases per 1 million of inhabitants, with the number of internet users a given country as a covariate. Estimates of functions slopes are given with standard errors (se), 95 % confidence intervals (LL - lower confidence intervals, UL - upper confidence intervals), *t* - test (with residual degree of freedom) and statistical significance (*p*). Model explained 29 % variation in data (adjusted R^2).

**Variable**	**estimate**	***se***	***LL***	***UL***	***t(16)***	***P***
(Intercept)	4.760	1.221	2.172	7.349	3.899	0.001
Cases per 1M in a country (log10 transformed)	-0.272	0.142	-0.574	0.030	-1.909	0.074
Number of internet users in a country (log10 transformed)	-0.348	0.145	-0.656	-0.039	-2.391	0.029

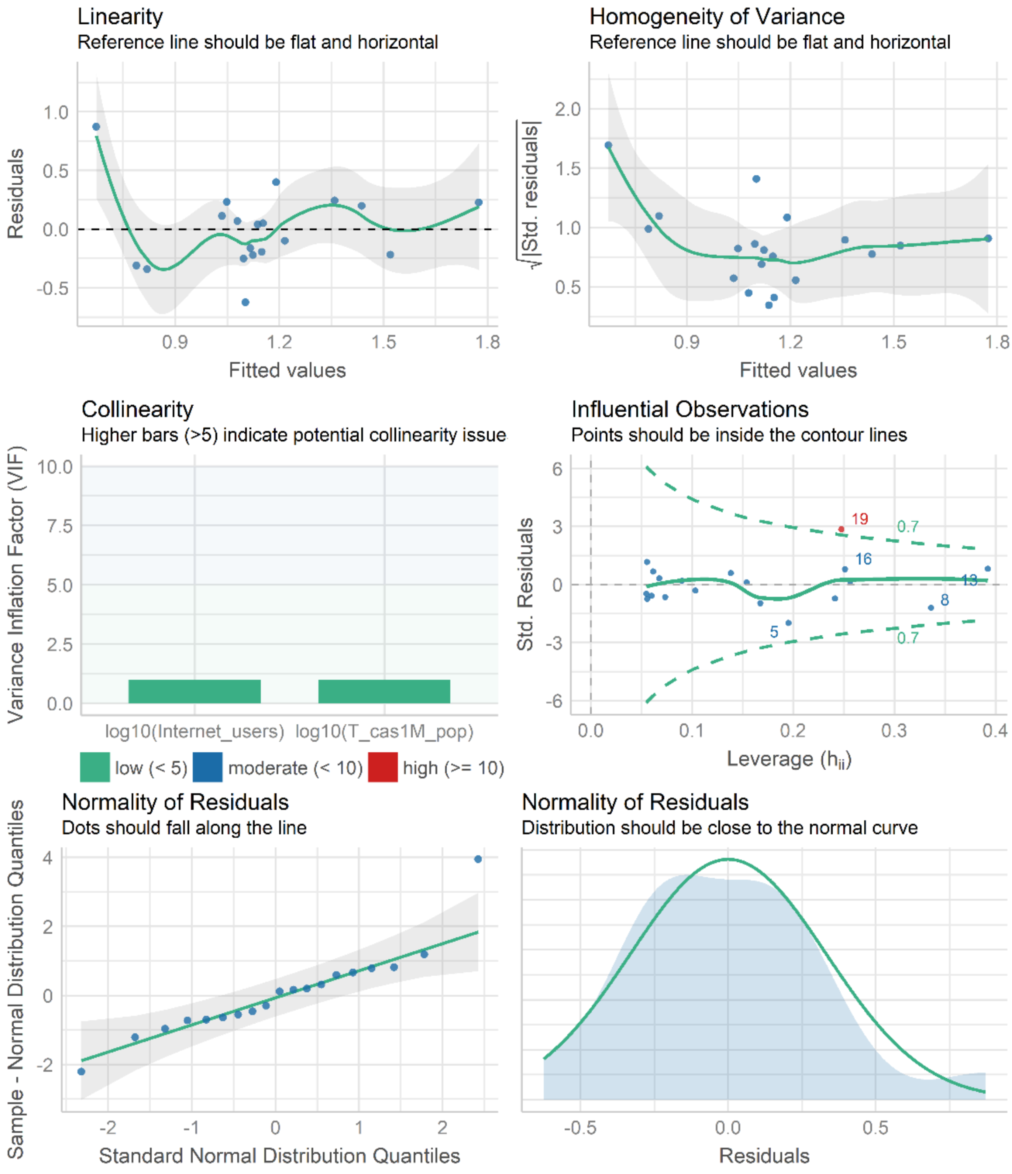

Figure S11. Tests of assumptions of the linear model for association between the relative interest in coronavirus memes (Google Trends index for string: “covid19 meme”) and total number of COVID-19 cases per 1 million of inhabitants, with the number of internet users in a given country as a covariate. Global test revealed no violation of assumptions of the linear model. Results of the model see: Table S6.

Search string: **"Covid meme"**

Table S7. Results of the general linear model testing the association between the occurrence of interest in coronavirus memes (Google Trends index for search string "covid meme") and total number of deaths due to COVID-19 per 1 million of inhabitants, and number of internet users in a given country as a covariate. Odds ratio (*OR*) with 95 % confidence intervals (LL - lower confidence intervals, UL - upper confidence intervals), Wald *z* - test (with residual degree of freedom) and statistical significance (*p*). Model explained 39 % variation in data (McFadden pseudo-R^2).

**Variable**	***OR***	***LL***	***UL***	***z(186)***	***P***
(Intercept)	<0.001	<0.001	0.000	-5.201	<0.001
Deaths per 1M in a country (log10 transformed)	2.844	1.369	6.841	2.570	0.010
Number of internet users in a country (log10 transformed)	8.215	3.611	22.859	4.528	<0.001

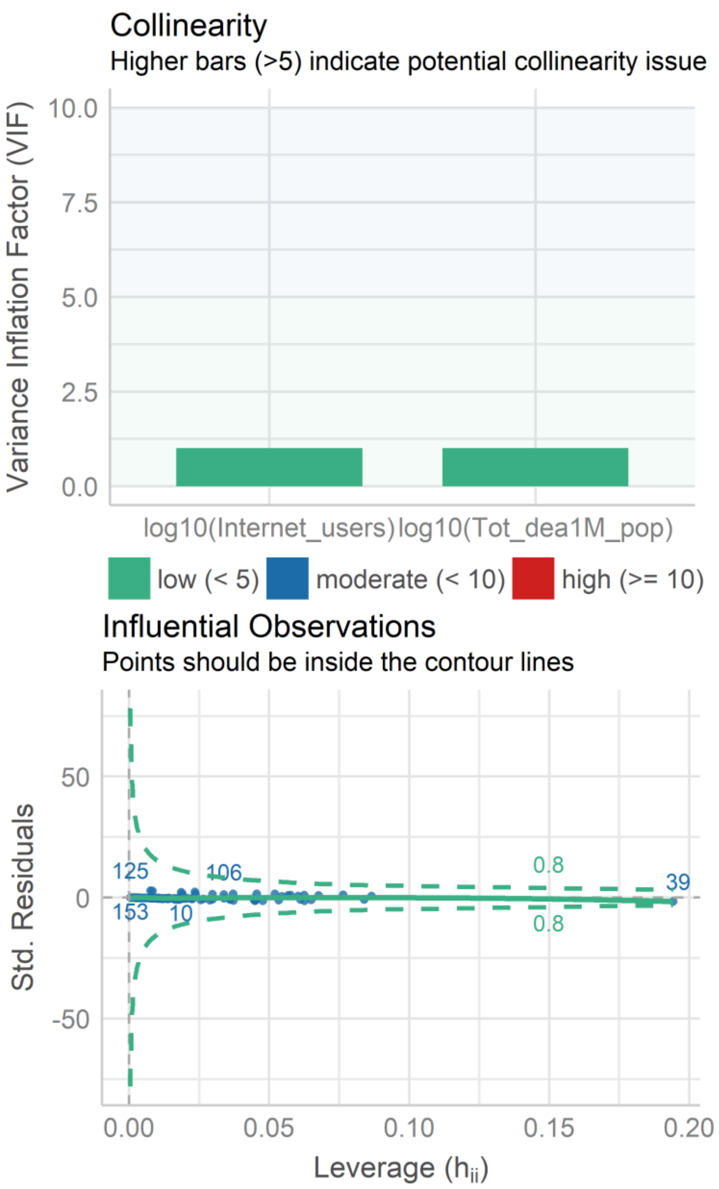

Figure S12. Tests of assumptions of the general linear model with the logit-link function and binomial error distribution, testing association between the occurrence of relative interest in coronavirus memes (Google Trends index for string: "covid19 meme") and total number of deaths due to COVID-19 per 1 million of inhabitants, with the number of internet users in a given country as a covariate. Results of the model see: Table S7.

Table S8. Results of the general linear model testing the association between the occurrence of interest in coronavirus memes (Google Trends index for search string “covid19 meme”) and total number of COVID-19 cases per 1 million of inhabitants, and number of internet users in a given country as a covariate. Odds ratio (*OR*) with 95 % confidence intervals (LL - lower confidence intervals, UL - upper confidence intervals), Wald *z* - test (with residual degree of freedom) and statistical significance (*p*). Model explained 44 % variation in data (McFadden pseudo-R^2).

**Variable**	***OR***	***LL***	***UL***	***z(209)***	***P***
(Intercept)	<0.001	<0.001	0.000	-4.916	<0.001
Cases per 1M in a country (log10 transformed)	5.215	1.907	18.353	2.887	0.004
Number of internet users in a country (log10 transformed)	2.910	1.951	4.879	4.631	<0.001

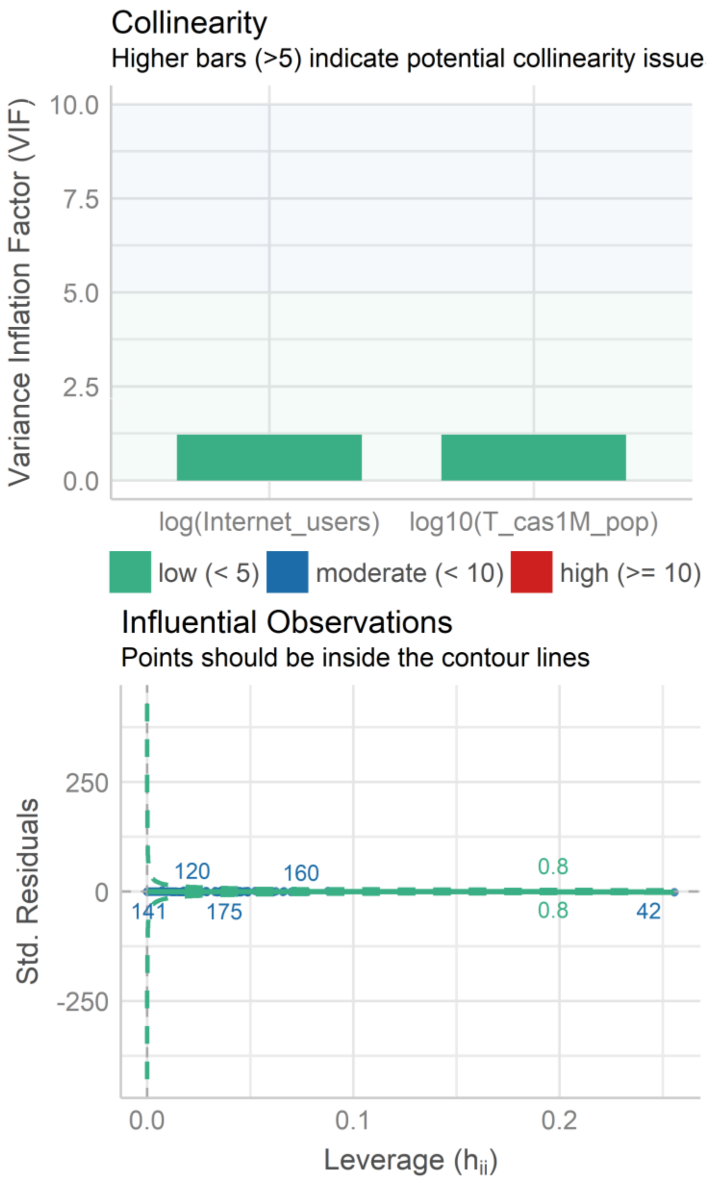

Figure S13. Tests of assumptions of the general linear model with the logit-link function and binomial error distribution, testing association between the occurrence of relative interest in coronavirus memes (Google Trends index for string: “covid19 meme”) and total number of COVID-19 cases per 1 million of inhabitants, with the number of internet users in a given country as a covariate. Results of the model see: Table S8.

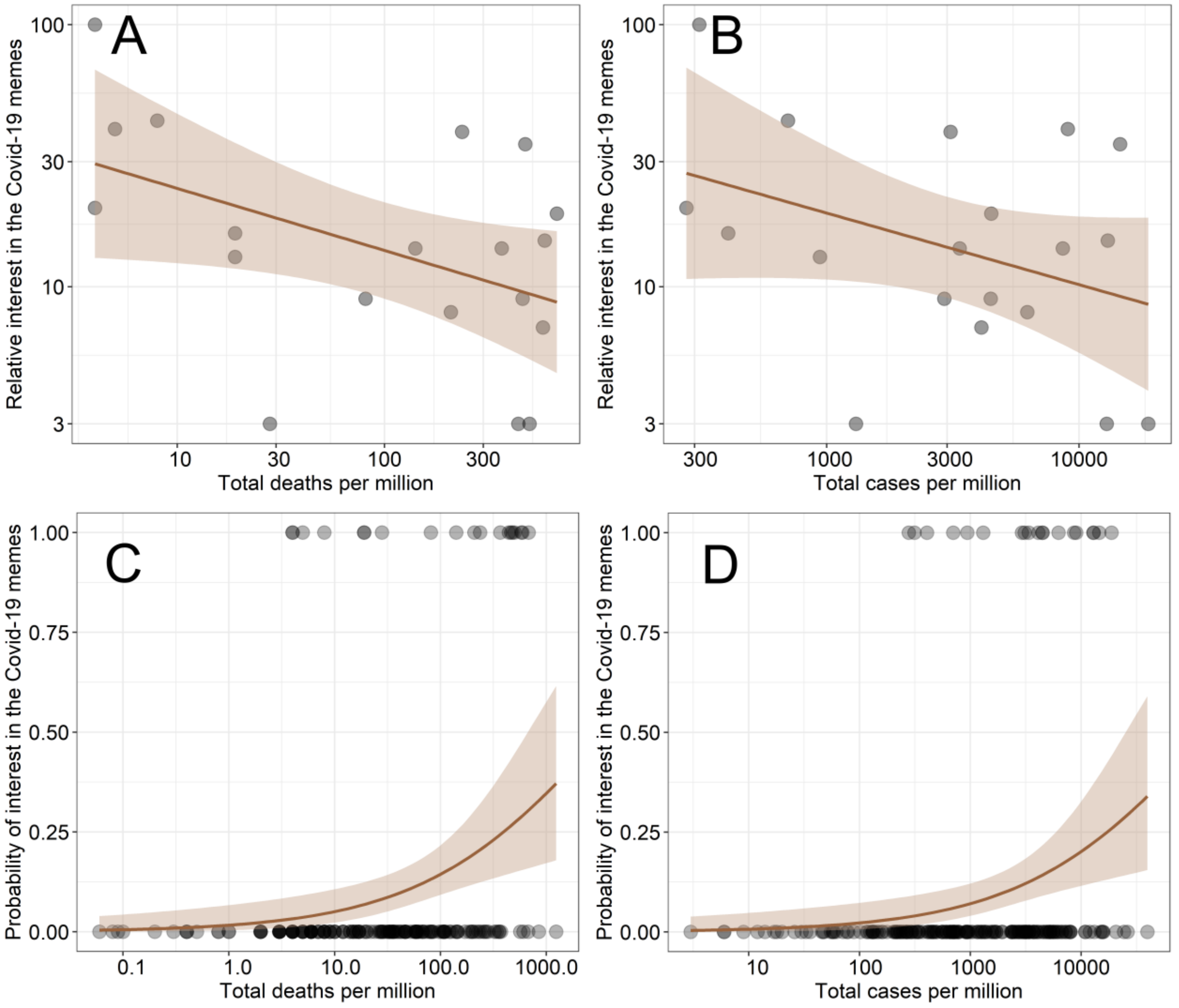

Figure S14. The associations between the interest in coronavirus memes and coronavirus statistics in different countries. The relative interest (measured as the Google Trends index for search string "covid19 meme") in coronavirus memes in different countries and its relation to (A) total number of deaths due to COVID-19 per 1 million inhabitants of a given country and (B) the total number of COVID-19 cases per 1 million inhabitants. The probability of occurrence of interest in coronavirus memes in different countries in relation to (C) the total number of deaths due to COVID-19 per 1 million inhabitants of a given country and (D) the total number of cases per 1 million inhabitants. For A-B a linear regression is fitted with 95% confidence interval, for C-D a logistic regression is fitted with 95% confidence interval. Data points are transparent for better visibility of overlapping values.

Results of cumulative link mixed model comparing funniness of the coronavirus memes, non-coronavirus memes and images being not memes. Analysis restricted to Polish respondents only.

Table S9. Results of the cumulative link mixed model comparing funniness of coronavirus memes with non-coronavirus memes and sample of images (not a meme). Analysis for Polish respondents only. The threshold coefficients show the transition chances in the questionnaire score point to the next. The model included effects of age, sex, and interaction between age and sex. Random factors were questionnaire identity and participants identity (nested in questionnaire identity). Odds ratio (*OR*) with 95 % confidence intervals (LL - lower confidence intervals, UL - upper confidence intervals), Wald *z* - test (with effective degree of freedom) and statistical significance (*p*). Model explained 15 % variation in data (McFadden pseudo-R^2).

**Variable**	***OR***	***LL***	***UL***	***z(11)***	***p***
Threshold coefficients:					
1|2	0.306	0.211	0.44	-6.226	<0.001
2|3	1.250	0.864	1.809	1.183	0.237
3|4	4.931	3.388	7.179	8.329	<0.001
4|5	23.947	15.937	35.982	15.287	<0.001
Fixed effects:					
Meme = Non coronavirus	0.459	0.388	0.543	-9.072	<0.001
Meme = Not meme	0.028	0.022	0.036	-28.051	<0.001
Age	0.600	0.422	0.853	-2.843	0.004
Gender = Male	1.136	0.686	1.880	0.495	0.621
Age*Gender = Male	1.923	1.188	3.112	2.660	0.008

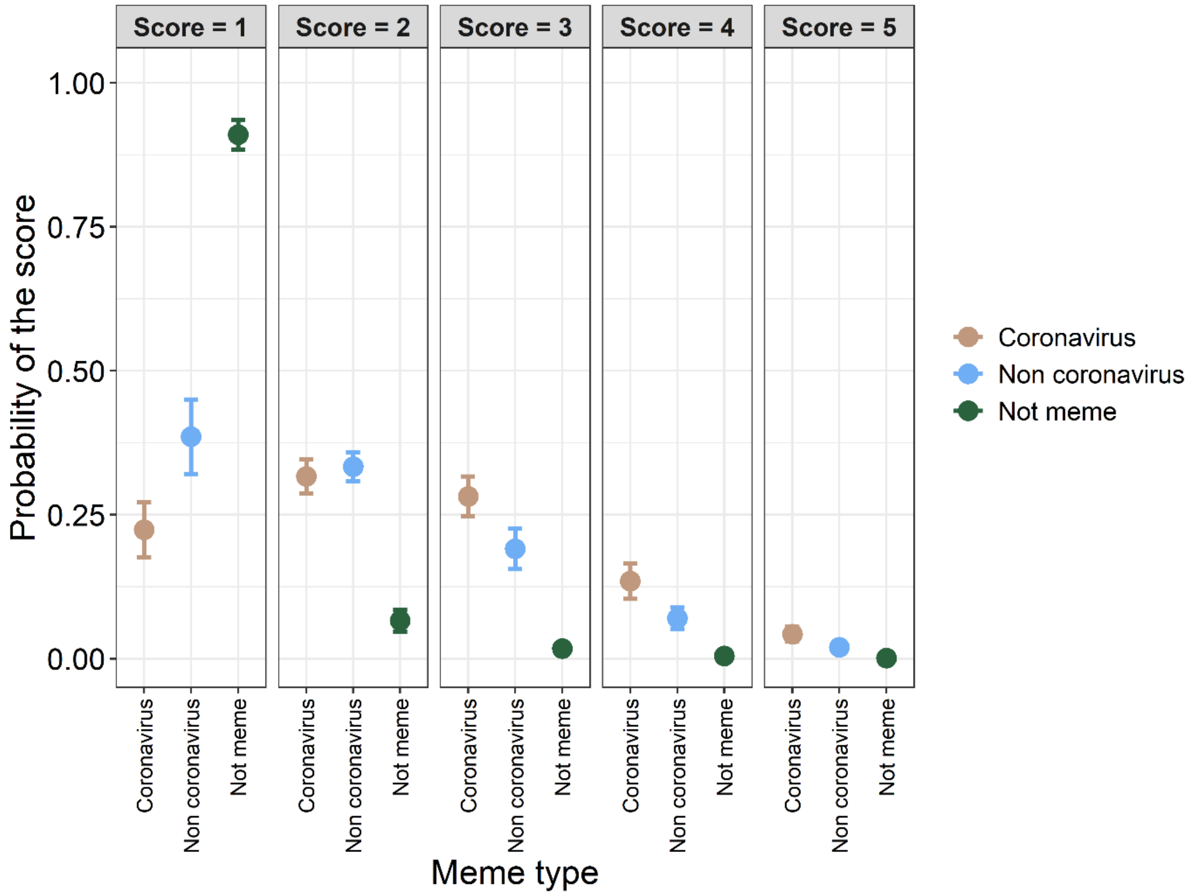

Figure S15. Predicted probability of the different questionnaire scores (grey facet labels) for coronavirus memes, non-coronavirus memes and sample of images (not a meme). The results from the cumulative link mixed model (Table S9) performed on data from Polish respondents only.

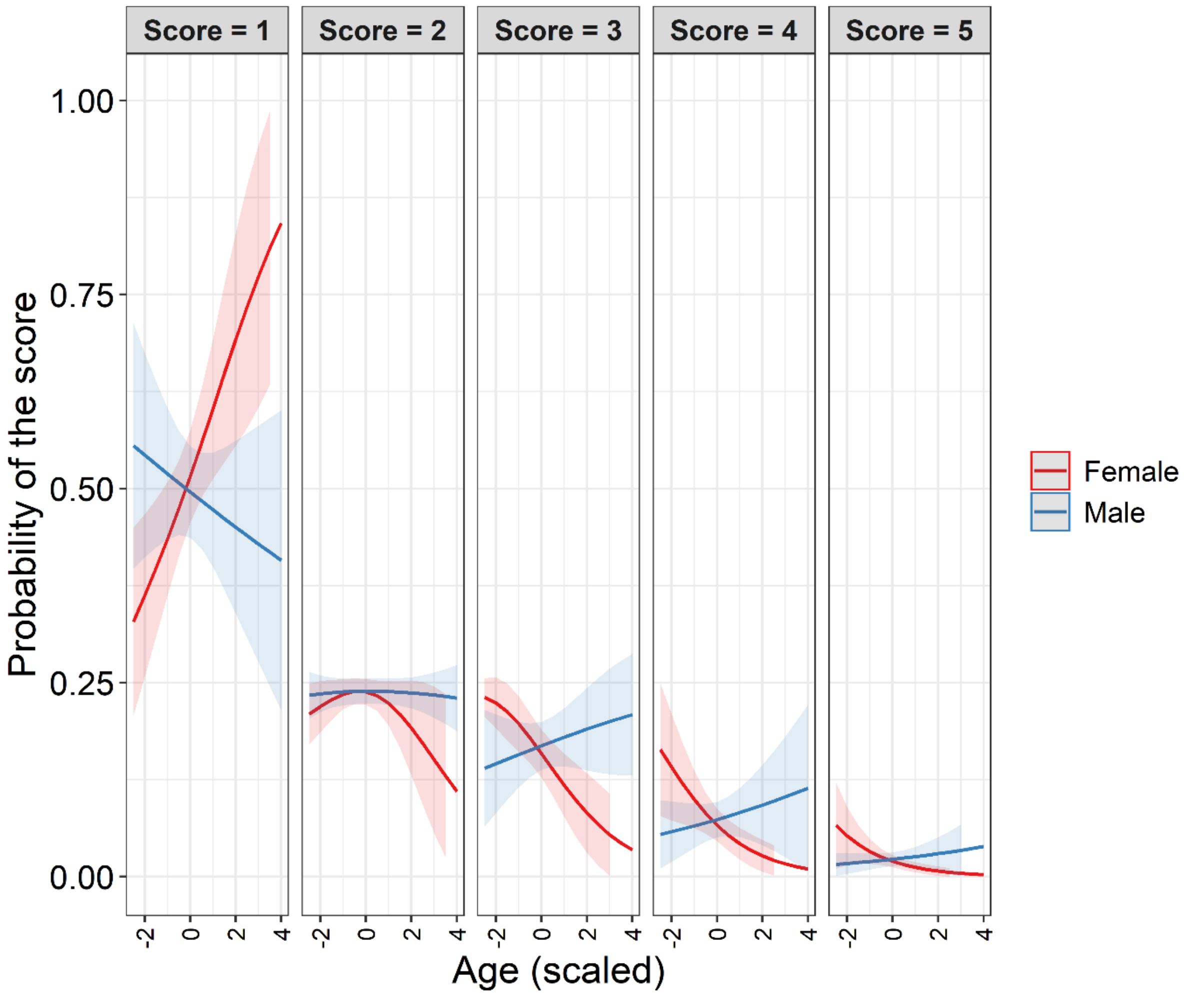

Figure S16. Predicted changes in the probability of the different questionnaire scores (grey facet labels) in two genders across age (scaled). The results from the cumulative link mixed model (Table S9) performed on data from Polish respondents only.
